# Key Regulators of Sucrose Metabolism Identified through Comprehensive Comparative Transcriptome Analysis in Peanuts

**DOI:** 10.3390/ijms22147266

**Published:** 2021-07-06

**Authors:** Weitao Li, Li Huang, Nian Liu, Manish K. Pandey, Yuning Chen, Liangqiang Cheng, Jianbin Guo, Bolun Yu, Huaiyong Luo, Xiaojing Zhou, Dongxin Huai, Weigang Chen, Liying Yan, Xin Wang, Yong Lei, Rajeev K. Varshney, Boshou Liao, Huifang Jiang

**Affiliations:** 1 Key Laboratory of Biology and Genetic Improvement of Oil Crops, Ministry of Agriculture, Oil Crops Research Institute of the Chinese Academy of Agricultural Sciences, Wuhan 430062, China; 82101171080@caas.cn (W.L.); huangli5100@126.com (L.H.); lnian0531@caas.cn (N.L.); ynchen126@126.com (Y.C.); guojianbin1990@163.com (J.G.); yubolun1990@163.com (B.Y.); huaiyongluo@caas.cn (H.L.); zhouxiaojing@caas.cn (X.Z.); dxhuai@caas.cn (D.H.); wgchen2015@163.com (W.C.); yanliying@caas.cn (L.Y.); wangxin456_2000@163.com (X.W.); leiyong@caas.cn (Y.L.); lboshou@hotmail.com (B.L.); 2 Center of Excellence in Genomics & Systems Biology, International Crops Research Institute for the Semi-Arid Tropics (ICRISAT), Hyderabad 502324, India; M.Pandey@cgiar.org (M.K.P.); R.K.Varshney@CGIAR.ORG (R.K.V.); 3 Oil Research Institute of Guizhou Province, Guizhou Academy of Agricultural Science, Guiyang 550006, China; chenglq2021@163.com; 4 State Agricultural Biotechnology Centre, Centre for Crop and Food Innovation, Murdoch University, Murdoch 6150, Australia

**Keywords:** transcriptional regulation, sucrose metabolism, RNA-based sequencing (RNA-seq), development, peanut seed

## Abstract

Sucrose content is a crucial indicator of quality and flavor in peanut seed, and there is a lack of clarity on the molecular basis of sucrose metabolism in peanut seed. In this context, we performed a comprehensive comparative transcriptome study on the samples collected at seven seed development stages between a high-sucrose content variety (ICG 12625) and a low-sucrose content variety (Zhonghua 10). The transcriptome analysis identified a total of 8334 genes exhibiting significantly different abundances between the high- and low-sucrose varieties. We identified 28 differentially expressed genes (DEGs) involved in sucrose metabolism in peanut and 12 of these encoded sugars will eventually be exported transporters (SWEETs). The remaining 16 genes encoded enzymes, such as cell wall invertase (CWIN), vacuolar invertase (VIN), cytoplasmic invertase (CIN), cytosolic fructose-bisphosphate aldolase (FBA), cytosolic fructose-1,6-bisphosphate phosphatase (FBP), sucrose synthase (SUS), cytosolic phosphoglucose isomerase (PGI), hexokinase (HK), and sucrose-phosphate phosphatase (SPP). The weighted gene co-expression network analysis (WGCNA) identified seven genes encoding key enzymes (CIN, FBA, FBP, HK, and SPP), three SWEET genes, and 90 transcription factors (TFs) showing a high correlation with sucrose content. Furthermore, upon validation, six of these genes were successfully verified as exhibiting higher expression in high-sucrose recombinant inbred lines (RILs). Our study suggested the key roles of the high expression of SWEETs and enzymes in sucrose synthesis making the genotype ICG 12625 sucrose-rich. This study also provided insights into the molecular basis of sucrose metabolism during seed development and facilitated exploring key candidate genes and molecular breeding for sucrose content in peanuts.

## 1. Introduction

Peanut or groundnut (*Arachis hypogaea* L.), an important oil seed crop, has been widely grown in more than 100 countries [1]. During 2018, the global planting area reached 28.52 million hectares and the annual production of peanut pods (with shells) was more than 45.95 million tonnes [1]. The seeds of peanut are not only rich in proteins (20–40%), lipids (40–60%), and carbohydrates (10–20%) [2] but also rich in vitamins, minerals, and antioxidants, and they can be directly eaten, boiled, roasted, or processed in candies, cookies, and chocolates [3]. Peanuts are considered as one of the favorite and important nuts in the market, and its consumption in food is on a continuous rise. Flavor is an important quality consideration for peanut in food uses because of consumer preference. High carbohydrate content was directly associated with increasing sweetness and superior flavor of seeds [4]. Sucrose is the major carbohydrate in peanut seeds, which comprised approximately 90% of the total sugars in peanut seeds and directly influenced the sweetness of raw peanuts [5]. Furthermore, sucrose could be hydrolyzed to fructose and glucose because of their water-soluble nature and could subsequently react with free amino acids to generate characteristic flavor during roasting [6]. In addition, sucrose content may play a significant role in the tolerance to low temperature and salt for plants [7,8,9]. Realizing the important role in quality and adaptation to abiotic stress in peanuts, it is essential to understand the molecular mechanism and identify key regulators of sucrose metabolism in peanuts.

Sucrose is the main product of photosynthesis exported from the leaves (source tissues) to different non-photosynthetic plant organs (sink tissues) based on the carbon needed for growth as well as the synthesis of storage reserves such as protein, starch, and oil [10]. The main metabolic pathways connecting carbon partitioning to the main storage compounds in seeds and key enzymatic steps involved in seed development are well characterized in the previous studies [11,12]. Photoassimilates from source tissues enter seed via the phloem. Sucrose plays a central role and is partitioned into different storage-specific pathways including the starch and sucrose metabolism pathway, the oxidative pentose phosphate pathway, and the glycolytic pathways, leading to the accumulation of storage reserves [12]. The tight regulation and changes in synthesis, transportation, and utilization of sucrose affect plant growth and development as well as sucrose accumulation. As one of the important quality traits, a large number of studies have focused on sucrose in recent years, especially in edible fruit or stalk, such as peaches [13,14], pears [15], apples [16,17], sugarcane [18], and sorghum [19,20]. The previous studies on plant sucrose metabolism reported a number of crucial enzymes such as sucrose synthase (SUS), sucrose phosphate synthase (SPS), sucrose-phosphate phosphatase (SPP), invertase (IN), fructose kinase (FK), and hexokinase (HK) [21]. The sugars will eventually be exported transporters (SWEET) gene family [22] includes important sugar transporter proteins, which had been reported playing a key role in the regulation of sucrose accumulation in *Arabidopsis* [23], soybean [24], and apples [17]. In addition, transcription factors such as WRKY [25], MYB [26], and bZIP [27] were likely to be involved in sucrose metabolism. Overall, the synthesis and accumulation of sucrose is a dynamic and complex process controlled by a regulatory network of multiple genes in plants. Although a large number of genes have been reported to participate in sucrose metabolism in *Arabidopsis* and fruits, the regulatory networks of sucrose metabolism in peanut seeds is still ambiguous. 

A couple of genetic studies on sucrose content in peanut seeds suggested its content being influenced by genotype, maturity duration, planting environments, and genotype-by-environment interaction [6,28]. Pattee et al. [28] identified large variation in sucrose content (20.3–42.2 mg/g) in peanut seeds and very high broad-sense heritability in a set of 52 genotypes belonging to three different market-types (Virginia, runner, and fastigiata). Another study based on diallel crosses involving four parents showed reciprocal cross differences for sucrose content in peanut seeds, indicating an important role of cytoplasmic genes [29].

The high-quality reference genome sequences of wild diploid progenitor species (*Arachis duranensis* and *A. ipanesis*), wild tetraploid (*A. monticola*), and both the subspecies (*hypogaea* and *fastigiata*) of cultivated tetraploid peanut (*A. hypogaea*) have become available recently [30,31,32,33,34,35,36]. The availability of these genome sequences is now facilitating precise structural and functional genomics studies in peanuts [37]. RNA sequencing technology provides an opportunity to dissect the regulatory network of sucrose metabolism during seed development in peanuts. In the previous study on the evaluation of sucrose content in peanut germplasm (unpublished), ICG 12625 was identified with high sucrose based on high-performance liquid chromatograph (HPLC) analysis. In this study, we performed an evaluation of sucrose content and a comprehensive comparative transcriptome at seven seed developmental stages between a high-sucrose content variety (ICG 12625) and a low-sucrose content variety (Zhonghua 10). This study targeted the discovery of differentially expressed genes related to sucrose metabolism and the construction of a regulatory network for sucrose metabolism in peanuts. In addition, it also provided new insights into the regulatory network underlying the sucrose content of peanut seed, which may be useful in the genetic improvement of flavor in peanuts.

## 2. Results

### 2.1. Significant Difference of Sucrose Content between ICG 12625 and Zhonghua 10 during Seed Development

**Figure 1:**
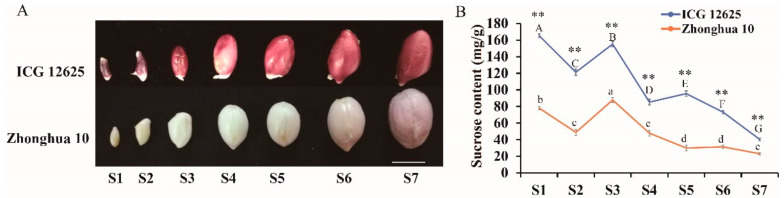
Phenotype differences between ICG 12625 and Zhonghua 10 at seven stages of development. (**A**) Seeds at seven different stages of development (S1–S7) in ICG 12625 and Zhonghua 10. The white solid line was a scale bar of 1 cm. (**B**) The changes in sucrose content in the seeds of two peanut varieties at different development stages (S1–S7). The value was an average sucrose content of three biological replications at each stage, and the error bar indicated standard error. The “**” represent extremely significant (*p* < 0.01) differences between the two varieties according to the independent samples *t*-test. Different capital or small letters indicate that the sucrose content in the developing seeds of a variety was significant different (*p* < 0.05) according to Bonferroni’s multiple comparison test.

A similar broken line graph of sucrose content in seeds showed a trend of up (S1) to down (S2) to up (S3), then it gradually decreased from S4 to S7 in ICG 12625 and Zhonghua 10 (Figure 1B). Among different seed development stages, the sucrose content was recorded high at S1 (168.1 mg/g in ICG 12625 and 78.3 mg/g in Zhonghua 10), declined at S2 (125.4 mg/g in ICG 12625 and 47.9 mg/g in Zhonghua 10), again increased at S3 (153.5 mg/g in ICG 12625 and 89.6 mg/g in Zhonghua 10), and finally declined to 40.8 mg/g in ICG 12625 and 21.4 mg/g in Zhonghua 10 at S7 (Figure 1B). The highest point appeared at S1 (168.1 mg/g) for ICG 12625 and S3 (89.6 mg/g) for Zhonghua10, which was about four-fold of the lowest value at the S7 stage either in both varieties (Supplementary Table S1). Although the two varieties displayed a similar variation trend in sucrose content during seed development, the sucrose content of ICG 12625 was always about two-fold higher than that of Zhonghua 10 at all seven stages (*p* < 0.01) (Figure 1B, Supplementary Table S1).

### 2.2. Transcriptome Profile of Seed Development in ICG 12625 and Zhonghua 10

**Figure 2:**
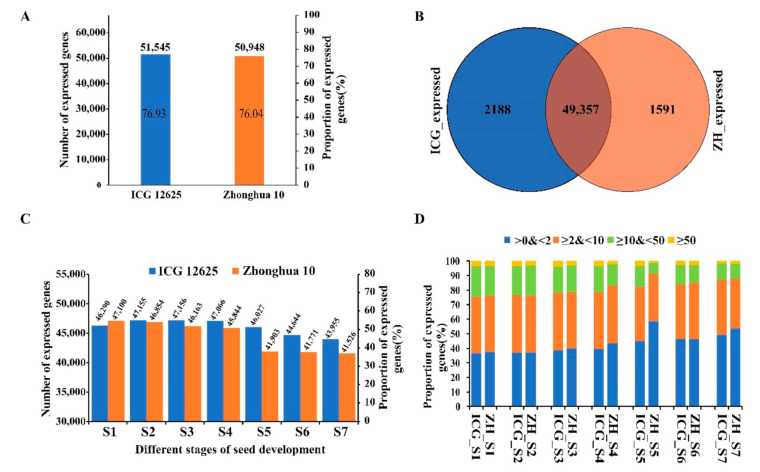
Gene expression levels during seed development in ICG 12625 and Zhonghua 10. (**A**) Total number and proportion of genes expressed in ICG 12625 and Zhonghua 10. (**B**) Venn diagram showed the intersection of genes expressed in ICG 12625 and Zhonghua 10. (**C**) The number and proportion of genes expressed at each stage of seed development in ICG 12625 and Zhonghua 10. (**D**) Fraction of genes expressed at different expression levels (based on FPKM) during seed development in ICG 12625 and Zhonghua 10.

A total of more than 0.25 billion high-quality reads (average ~12 million reads for each sample) were generated for each variety (Supplementary Table S2). After mapping the reads to the cultivated peanut reference genome, eventually, a total of 51,545 and 50,948 genes were identified and found expressed in seeds of ICG 12625 and Zhonghua 10 covering 76.93% and 76.04% of all the annotated genes on the whole genome, respectively (Figure 2A). Among these expressed genes, 49,357 genes were detected in both varieties, and few genes were specifically expressed (2188 genes in ICG 12625 and 1591 genes in Zhonghua 10) (Figure 2B). The number of expressed genes gradually decreased during seed development, which ranged from 47,156 (70.38%, S3) to 43, 955 (65.60%, S7) in ICG 12625 and 47,100 (70.29%, S1) to 41,526 (61.97%, S7) in Zhonghua 10 (Figure 2C). The number of expressed genes in ICG12625 was higher than those in Zhonghua10 across stages, except for S1 (Figure 2C). The proportion of genes with different expression levels was similar at each stage in ICG 12625 and Zhonghua 10, except at S5 stage (Figure 2D). The high repeatability of the transcriptome data was observed among the biological replicates with a value of above 0.8 for the Spearman correlation coefficient (Supplementary Figure S1).

### 2.3. Stage-Specific Transcriptome Profile during Seed Development in ICG 12625 and Zhonghua 10

**Figure 3:**
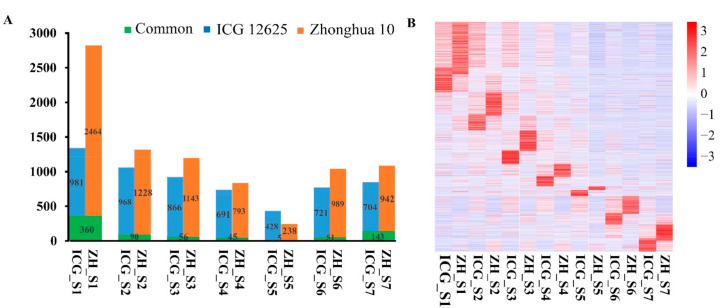
Stage-specific expressed genes during seed development in ICG 12625 and Zhoanghua 10. (**A**) The number of stage-specific expressed genes specifically and commonly in ICG 12625 and Zhonghua 10 at each stage of seed development. (**B**) Heatmap showing the expression profile of stage-specific expressed genes in ICG 12625 and Zhonghua 10 during seed development. Color scale represented the Z-score.

PCA revealed a different development profile of seed tissues between ICG 12625 and Zhonghua 10 varieties (Supplementary Figure S2). Based on the stage specificity (SS) algorithm, 6109 and 8547 genes were found specifically expressed at a particular stage in ICG 12625 and Zhonghua 10, respectively. The number of genes with stage-specific expression varied from 433 (S5) to 1341 (S1) for ICG 12625 and 243 (S5) to 2824 (S1) for Zhonghua 10 (Figure 3A). A set of 750 stage-specific genes commonly expressed in both the varieties ranging from 5 (S5) to 360 (S1) (Figure 3A). A heatmap of the stage-specific gene expression indicated that the most stage-specific genes were expressed at the early stage (S1–S3, Figure 3B). The number of stage-specific genes were detected less in ICG 12625 as compared to Zhonghua 10 across stages, except S5. Interestingly, GO analysis indicated that the stage-specific genes expressed in ICG 12625 10 or Zhonghua acted in the same metabolic process during seed development (Supplementary Figure S3). A total of 137 genes including 30 SWEET genes and 107 key enzyme genes involved in sucrose metabolism were identified. The similar expression patterns of these genes indicated that the similar molecular event occurred in both varieties (Supplementary Figure S4). In particular, 26 genes of them showed stage-specific expression (Supplementary Table S3). For example, gene *arahy.2I1JD4* was specifically expressed at the S3 stage in ICG 12625, whereas gene *arahy.T958MW* was specifically expressed at the S7 stage in Zhonghua 10.

### 2.4. Differential Gene Expression between ICG 12,625 and Zhonghua 10

**Figure 4:**
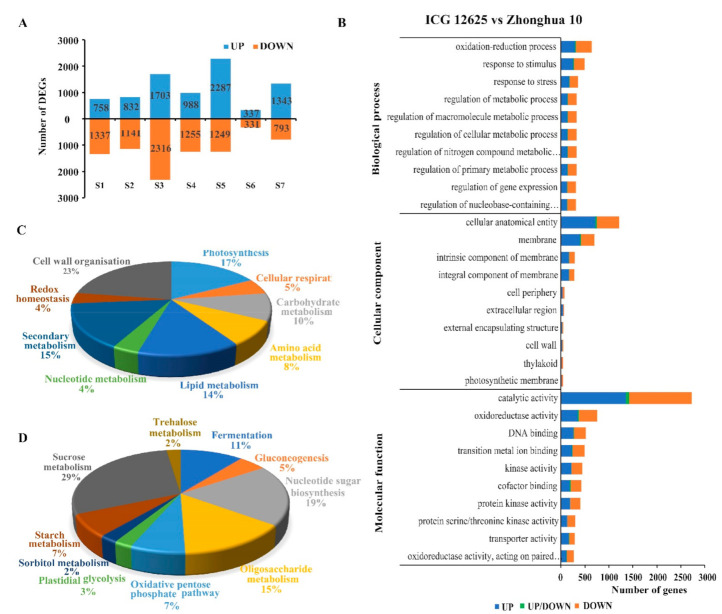
Differentially expressed genes between ICG 12625 and Zhonghua 10 at seven stages of seed development. (**A**) The number of upregulated (upper bars) and downregulated (lower bars) genes in ICG 12625 as compared with Zhonghua 10 at each stage of seed development. (**B**) Gene ontology analysis of DEGs. “UP/DOWN” means the genes were upregulated at one stage of seed development and downregulated at another stage of seed development. (**C**) The pie chart showing the proportion of genes involved in different metabolic pathways. (**D**) The pie chart showing the proportion of genes involved in carbohydrate metabolism.

A total of 8334 DEGs were identified based on the standard described in the section of material and methods. Compared with the low-sucrose content variety (Zhonghua 10), 4416 genes including 270 TFs showed significant up-regulation, and 3774 genes including 319 TFs showed down-regulation at seven developmental stages in the high-sucrose variety (ICG 12625) (Supplementary Figure S5A). Another 144 DEGs including 14 TFs showed variable expression patterns between the two varieties at seven stages (Supplementary Figure S5A). For example, *arahy.H8DQQQ* was found up-regulated at S1–S3 and down-regulated at S4–S7. The number of up-regulated and down-regulated genes throughout the seed development in ICG 12625 was 166 and 175, respectively (Supplementary Figure S5B). The most DEGs (4019) were detected at the S3 stage followed by the S5 stage (3536) between the two varieties (Figure 4A).

GO analysis indicated that these DEGs were mainly involved in catalytic activity (GO:0003824, 2728 genes), cellular anatomical entity (GO:0110165, 1211 genes), and oxidoreductase activity (GO:0016491, 757 genes) during the complete seed development process (Figure 4B). To further overview the metabolic pathway differences between two varieties with different sucrose content, all the DEGs were overlaid onto the available metabolic pathways using the MapMan tool, and more than 10% of genes were annotated to known metabolic pathways. These DEGs mainly participated in cell-wall organization, the photosynthesis process, and secondary metabolism at either the whole seed developmental process or each stage of development (Figure 4C, Supplementary Table S4). Based on the metabolism pathway analysis results at each stage of DEGs, a total of 81 genes were identified participating in carbohydrate metabolism. It mainly included sucrose metabolism, nucleotide sugar biosynthesis, and oligosaccharide metabolism (Figure 4D). Two genes (*arahy.EMU471* and *arahy.VIF38V*) encoding fructose-1,6-bisphosphatase (FBP) were found involved in both gluconeogenesis and sucrose metabolism.

To validate the repeatability and reproducibility of RNA-seq data, 14 DEGs were selected to perform biologically independent qRT-PCR at seven stages in ICG 12625 and Zhonghua 10, respectively. The gene-specific primer pairs are listed in Supplementary Table S5. The similar expression patterns and linear regression analysis based on the transcriptome data and qRT-PCR results indicated a fine repeatability and good reliability between the RNA-seq data and the qRT-PCR results (Supplementary Figure S6).

### 2.5. DEGs Related to Seed Sucrose Metabolism between ICG 12625 and Zhonghua 10

**Figure 5:**
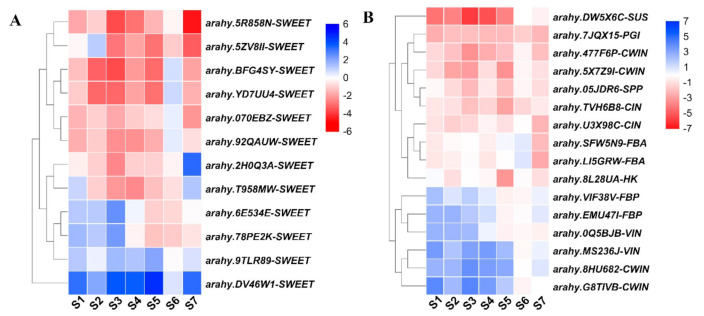
Expression comparison of genes related to sucrose metabolism in two varieties. The red and blue color indicate up-regulation and down-regulation in ICG 12625, respectively. (**A**) The heatmap showing the log2FC of 12 SWEET genes (**B**) The heatmap showing the log2FC of 16 enzyme genes.

Based on DEGs analysis, a total of 12 genes encoding SWEETs were identified (Supplementary Table S6, Figure 5A). The phylogenetic analysis revealed these SWEET genes were homologous to SWEET genes of *Arabidopsis* (Supplementary Figure S7). For example, three of them, *arahy.2H0Q3A*, *arahy.YD7UU4*, and *arahy.BFG4SY*, belonging to the same group, were homologous to AtSWEET15 (Supplementary Figure S7). Among them, the expression of eight SWEETs, including *arahy.5R858N*, *arahy.5ZV8II*, *arahy.BFG4SY*, *arahy.YD7UU4*, *arahy.070EBZ*, *arahy.92QAUW*, *arahy.2H0Q3A*, and *arahy.T958MW* were up-regulated in at least five developmental stages in ICG 12625. In particular, *arahy.5R858N*, *arahy.2H0Q3A*, *arahy.YD7UU4*, and *arahy.BFG4SY* were 10-fold higher during at least one stage in ICG 12625 than those in Zhonghua 10 (Figure 5A). Two SWEET genes (*arahy.6E534E* and *arahy.78PE2K*) were down-regulated at early seed development stages and up-regulated at late seed developmental stages. The remaining genes (*arahy.9TLR89* and *arahy.DV46W1*) were down-regulated throughout seven seed developmental stages.

Among 81 DEGs involved in carbohydrate metabolism, 16 genes participated in sucrose biosynthesis and degradation (Supplementary Table S6, Figure 5B). According to the gene function annotations, these genes encode CWIN (four genes), VIN (two genes), CIN (two genes), FBA (two genes), FBP (two genes), SUS (one gene), PGI (one gene), HK (one gene), and SPP (one gene). Five genes involved in sucrose biosynthesis, *arahy.7JQX15* (PGI), *arahy.05JDR6* (SPP), *arahy.8L28UA* (HK), *arahy.SFW5N9* (FBA), and *arahy.LI5GRW* (FBA), were highly expressed in ICG 12625 (Figure 5B). Similarly, *arahy.DW5X6C* encoding SUS, which catalyzed the reversible conversion of sucrose, was highly expressed in ICG 12625 (Figure 5B). Invertase (INV), including VIN, CWIN, and CIN, which catalyzed an irreversible breakdown of sucrose into hexose, showed different expression patterns between Zhonghua 10 and ICG 12625. *Arahy.0Q5BJB* and *arahy.MS236J* encoding VIN exhibited higher expression in Zhonghua 10, whereas two CINs (*arahy.U3X98C* and *arahy.TVH6B8*) exhibited higher expression in ICG 12625 (Figure 5B). Among four genes encoding CWIN, *Arahy.477F6P* and *arahy.5X7Z9I* were found highly expressed in ICG12625, whereas *arahy.8HU682* and *arahy.G8TIVB* genes showed high expression in Zhonghua 10 (Figure 5B). These results suggested that the differential expression of key SWEET and enzyme genes may be responsible for the different sucrose content in peanuts.

### 2.6. SWEET and Key Enzyme Genes Involved in the Regulatory Network of Sucrose Metabolism

**Figure 6:**
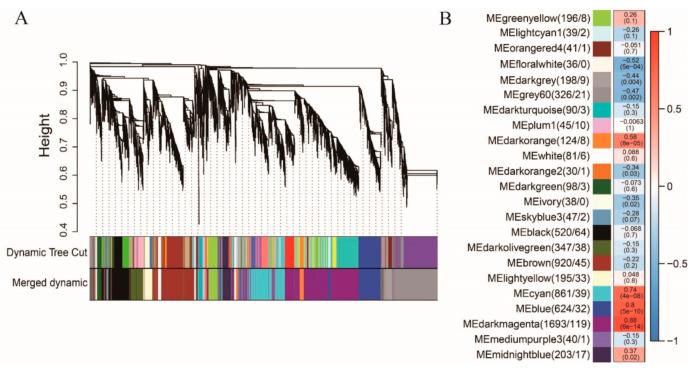
Co-expression network and correlations between module eigengene and sucrose content in peanut varieties. (**A**) Hierarchical clustering tree (dendrogram) of genes based on co-expression network analysis in ICG 12625 and Zhonghua 10. (**B**) Correlation analysis between expression pattern of genes in the 23 co-expression modules and sucrose content in two varieties. Boxes contained Pearson correlation coefficients and *p* values. The deeper red and blue color indicated that the ME of the module had more strong positive correlation and negative correlation with sucrose content, respectively. The number of genes/TFs in the module was provided on the left.

**Figure 7:**
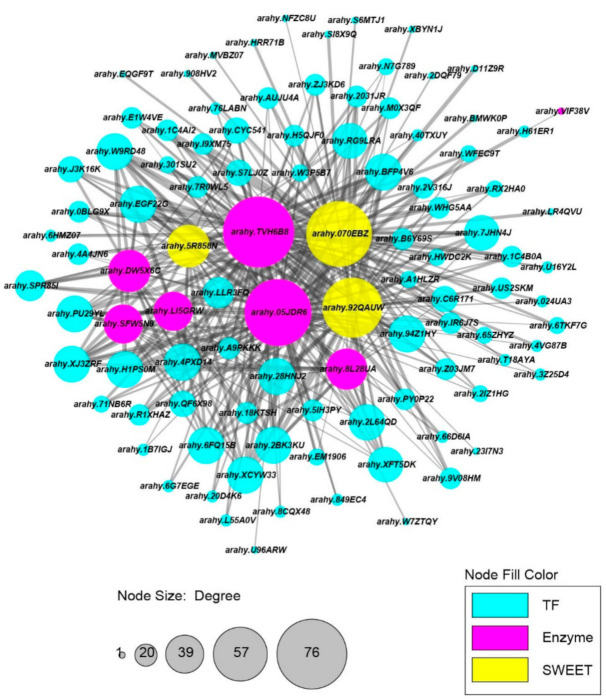
A gene co-expression network of 7 enzymes, 3 SWEETs genes, and 90 TFs. Each node represents a gene in the network and is labeled with the gene name. Fuchsia, yellow, and cyan nodes represent enzymes, SWEETs and TFs, respectively. Node size represents total connectivity. An edge indicates significant co-expression between two connected genes (weight value > 0.2).

**Figure 8:**
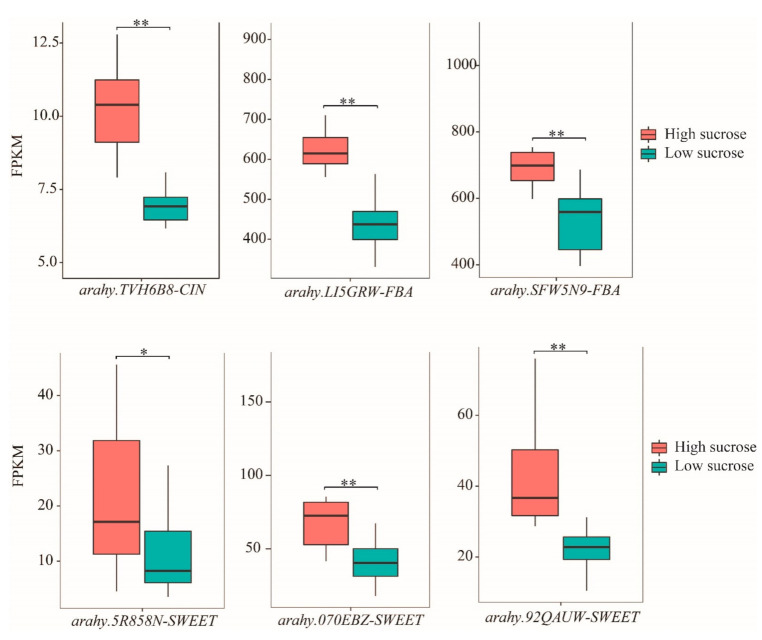
Expression level of six genes in the RIL lines with high sucrose and low sucrose. In each box, centerline shows the median; box limits indicate the 25th and 75th percentiles; whiskers extend 1.5 times the interquartile range from the 25th and 75th percentiles. Error bars represented standard errors (*n* = 12). Within plots, the “*” and “**” indicates statistically significant differences at *p* < 0.05 and *p* < 0.01 according to independent samples *t*-test, respectively.

To construct a sucrose-content-related gene regulatory network (GRN), the DEGs were selected to perform WGCNA after filtering. Finally, 6792 genes including 462 TFs were used for construction of a co-expression network leading to identification of 23 modules (Figure 6A). The number of genes among 23 modules ranged from 30 to 1693. The correlation coefficients between module eigengenes and sucrose content varied widely from 0.05 to 0.88, and the darkmagenta module was extremely significantly positive with sucrose content (r = 0.88, *p*-value < 0.001, Figure 6B). In the darkmagenta module, five SWEETs and seven genes including *arahy.TVH6B8* (CIN), *arahy.LI5GRW* (FBA), *arahy.SFW5N9* (FBA), *arahy.VIF38V* (FBP), *arahy.8L28UA* (HK), *arahy.05JDR6* (SPP), and *arahy.DW5X6C* (SUS) related to sucrose metabolism were identified as well as 119 TFs. A GRN of sucrose metabolism was constructed and visualized (weight value > 0.2, Figure 7) including seven sucrose-related enzymes, three SWEET genes (*arahy.5R858N*, *arahy.070EBZ*, and *arahy.92QAUW*), and 90 TFs. Based on the results provided by the Plant Regmap, the TFs belonging to ERF, MYB, bZIP, bHLH, NAC, and WRKY were identified with more binding sites to the promoter of seven sucrose-related enzymes and three SWEETs (Supplementary Table S7). In the previous study [38], the expression level of these seven sucrose-related enzymes and three SWEETs were evaluated in 24 RILs deriving from Zhonghua 10 and ICG 12625 at the early stage. The independent samples *t*-test analysis indicated that six genes including *arahy.TVH6B8* (CIN), *arahy.LI5GRW* (FBA), *arahy.SFW5N9* (FBA), *arahy.5R858N* (SWEET), *arahy.070EBZ* (SWEET), and *arahy.92QAUW* (SWEET) exhibited significantly higher gene expression in 12 high-sucrose content lines than that in 12 low sucrose content lines (Figure 8, *p* < 0.05). These results indicated that these six genes may highly associate with sucrose content in peanut seed.

## 3. Discussion

Sucrose content is an important factor determining quality and flavor of peanut seed, which directly affects the consumer’s preference. In this study, the dynamics of sucrose content with seed development in peanut were similar to those in vegetable soybean seed [39], indicating that the sucrose content was negatively correlated with the maturity of the seed (Figure 1). The sucrose content was high at an early stage in both varieties, and it was about two-fold higher in ICG 12625 than that in Zhonghua 10 throughout seed development. It is reasonable to mention that the final sucrose content in seeds is determined at the early stage (S1 to S3). Although ICG 12625 and Zhonghua 10 belong to different biological types of peanut, they exhibited a similar expression patterns of 137 genes related to sucrose metabolism (Supplementary Figure S4), indicating a general molecular basis of sucrose metabolism in seed development in these peanut varieties.

Sucrose is the main product generated during photosynthesis in leaves (source organ) and is later transported to seeds for storage in the form of carbohydrates, lipids, and protein [40]. The seed coat controls the rate of delivery of sucrose uptake into the seed by sucrose transporters and hexose transporters [12]. Sucrose plays a central role and is partitioned into different storage-specific pathways by multiple enzymes, leading to the accumulation of oil and proteins [41,42]. Sucrose can be degraded by CWIN and SUS, releasing hexose phosphates into the cytosol. Hexose can be further utilized through the oxidative pentose phosphate pathway and the glycolytic pathways, providing precursors for fatty acids and proteins. It should be noted that the metabolic intermediate phosphoenol pyruvate plays key roles in directing carbon partitioning toward protein and oil accumulation [12]. With the development of seeds, we found that the oil content and the protein content increased gradually (Supplementary Figure S9), and the change curve was opposite to that of sucrose content (Figure 1). The protein content of Zhonghua 10 was always higher than that of ICG 12625 at the S3–S7 stage of seed development (Supplementary Figure S9). The oil content of Zhonghua 10 was higher at the S3 and S4 stages of seed development compared with ICG12625, whereas there was no significant difference at the S5–S7 stages of seed development (Supplementary Figure S9). The ratios of protein–sucrose, oil–sucrose, and oil–protein increased as maturity approached (Supplementary Figure S10). The increase in the oil–sucrose ratio and the protein–sucrose ratio were due to a decrease in sucrose content and a rise in oil and protein content, respectively. The rise of the oil–protein ratio indicated that the increasing rate of oil content was faster than that of protein content. These results were consistent with the previous study [43]. Hence, most of the reduced sucrose may be converted into oil or protein during seed development in peanuts. The difference in sucrose content between ICG 12625 and Zhonghua 10 may have resulted in the difference in protein content (S3–S7) and oil content (S3 and S4).

The higher sucrose content in ICG12625 was already evident at stage S1 and might be related to amino acid metabolism and lipid metabolism based on the above analysis. Although the data of the protein content and the oil content of S1–S2 were missing, we can speculate that the protein content and the oil content of Zhonghua 10 at S1–S2 were higher than those of ICG 12625. The detailed analysis of DEGs at the S1 stage further supports the above speculation. From Supplementary Figure S11, we found many DEGs at the S1 stage involved in lipid metabolism and amino acid metabolism. Moreover, most of these genes exhibited higher expression levels in Zhonghua 10 than those in ICG 12625, which might be responsible for the higher protein and oil content in Zhonghua 10. For example, the transcripts of all seven genes involved in fatty acid biosynthesis showed higher expression levels in Zhonghua 10 at the S1 stage. Fifteen of the 18 genes that participated in amino acid biosynthesis were highly expressed in Zhonghua 10. In addition, the S4–S5 transition appears to be a significant developmental point (Figure 1B). We found different gene expression patterns in metabolic pathways between S4 and S5 (Supplementary Figure S11). During the transition from S4 to S5, in addition to the differences in amino acid metabolism, carbohydrate metabolism, and lipid metabolism between ICG 12625 and Zhonghua 10, light reactions and the Calvin cycle also showed great differences (Supplementary Figure S11). Most of the DEGs involved in light reactions and the Calvin cycle of the S4 stage showed high transcriptional activity in Zhonghua 10, whereas DEGs at the S5 stage showed the opposite pattern (Supplementary Figure S11). Hence, these genes might be responsible for the differences in S4–S5 transition between ICG 12625 and Zhonghua 10.

As a newly discovered sugar transporter in recent years, SWEET could not only transport sugars but also influence growth and development by regulating translocation efficiency of sugar across the membrane [44]. In *Arabidopsis*, the SWEET gene family of sugar transporters was divided into four different subclades (clade I-IV), in which various SWEET transporters preferentially transport distinct sugar moieties [22,44]. Clades I, II, and IV appeared to be predominantly hexose transporters, whereas clade III SWEETs transported predominantly sucrose although they also could transport hexoses [45]. SWEET transporters performed their functions at distinct cellular compartments. For example, with SWEET1, 8, 9, 11, 12, and 15 mainly localized in the plasma membrane [22,23,46,47,48,49], and SWEET9 and 15 localized in the Golgi [23,47]. In this study, a high correlation between expression of SWEET and sucrose content was also observed. A total of 12 SWEETs belonging to three clades (Supplementary Figure S7) were differentially expressed between ICG12625 and Zhonghua 10, of which eight SWEET genes were highly expressed across different developmental stages in ICG 12625 (Figure 5, Supplementary Table S6). Five SWEET genes (*arahy.BFG4SY*, *arahy.YD7UU4*, *arahy.2H0Q3A*, *arahy.5R858N*, and *arahy.5ZV8II*) belonging to clade III were homologous to gene *AtSWEET15* and *GmSWEET15* (Supplementary Figure S7), which positively regulated sucrose accumulation at expression level in *Arabidopsis* [50] and soybean [24]. Notably, in soybean, the overexpression of *GmSWEET15* increased sucrose content, meanwhile the knockout mutants greatly reduced sucrose content in the embryo [24]. Three genes, *arahy.070EBZ*, *arahy.92QAUW*, and *arahy.T958MW*, belonging to clade I showed higher expression in ICG 12625 compared with Zhonghua 10, whereas *arahy.6E534E* and *arahy.78PE2K* belonging to clade II showed the opposite expression pattern. Both clade I and II genes had been reported to be mainly involved in the transport of hexose between different cellular compartments [22,23,44,45] and may cause the change in sucrose/hexose, which was considered a signal that can regulate the accumulation of stored substances such as protein in plant seed during development [51,52]. The remaining two SWEET genes, *arahy.DV46W1* and *arahy.9TLR89*, were homologous to *AtSWEET11* and *AtSWEET12* in *Arabidopsis* (Supplementary Figure S7) and exhibited higher expression in Zhonghua 10 compared with ICG 12625. *AtSWEET11* and *AtSWEET12* were highly expressed in *Arabidopsis*-developing seeds and were responsible for the transportation of sucrose from the seed coat to the embryo [23]. In tomato, *SlSWEET12c* is highly expressed in tomato seeds and may facilitate sucrose unloading from the phloem down a concentration gradient maintained by CWIN to support seed filling [53]. Overall, it could be deduced that the high expression of SWEET genes, especially genes belonging to clade III, were partially responsible for the higher sucrose content in seeds of ICG 12625. Although there were two genes (*arahy.DV46W1* and *arahy.9TLR89*) belonging to clade III with higher expression in Zhonghua 10 compared with ICG 12625, their absolute value of gene expression was very low in both of the two varieties (Supplementary Figure S8).

Being one of the important elements of physiological and biochemical activities, sucrose also experiences a very complicated metabolism process in seed development [42,51,54,55]. A total of 16 key genes related to sucrose metabolism were identified encoding INV, SUS, FBP, SPP, FBA, HK, and PGI. It seems that ICG 12625 had more abundant up-regulated genes involved in both the biosynthesis and the degradation of sucrose, which resulted in the more vigorous metabolism and the accumulation of a high content of sucrose in the seed of ICG 12625 (Supplementary Figure S8). The previous studies revealed that the sucrose content was regulated positively (synthesis) and negatively (degradation) by genes involved in sucrose metabolism [56,57]. For the synthesis of sucrose, it had been reported that SPP [15], FBA [58], PGI [59], and HK [60] participated in sucrose synthesis and that their up-regulated expression could promote sucrose accumulation. In the present study, genes *arahy.7JQX15* (PGI), *arahy.05JDR6* (SPP), *arahy.8L28UA* (HK), *arahy.SFW5N9* (FBA), and *arahy.LI5GRW* (FBA), exhibited high levels of expression in ICG 12625, which, in turn, promoted the synthesis of sucrose and contributed to the final sucrose content in the peanut seed. For the degradation of sucrose, INV, including CWIN, VIN, and INV(CIN), irreversibly hydrolyzes sucrose producing glucose and fructose and is a key enzyme of carbon metabolism in sink tissues [21,61]. In this study, *arahy.0Q5BJB* and *arahy.MS236J* encoding VIN exhibited low expression in ICG 12625, indicating that the low intensive degradation of sucrose was possible, which is also a factor for the high sucrose accumulation in ICG 12625. In rice, the decreased expression of OsVIN2 could significantly increase the sucrose content in seed [62]. Although two CINs, *arahy.TVH6B8* and *arahy.U3X98C*, exhibited higher relative expression in ICG 12625, the absolute expression of these two genes was low, indicating that these two genes were not responsible for sucrose accumulation. It had been proved that CINs play an important role in the regulation of growth and cell development [63] with cellulose biosynthesis at low level of expression [64]. Interestingly, there were two CWIN genes showing opposite expression patterns with each other in ICG 12625 (*arahy.477F6P* and *arahy.5X7Z9I*) and Zhonghua 10 (*arahy.8HU682* and *arahy.G8TIVB*). CWIN could promote the transportation of sucrose from the source organ to the sink organ, prompting that it played various roles especially in reproduction and seed development [65,66]. The expression pattern of CWIN perhaps indicated a different molecular character in the utilization of sucrose, but without contribution to sucrose accumulation between ICG 12625 and Zhonghua 10. The degradation of sucrose was mainly catalyzed by SUS in sink tissues, and SUS activity was an indicator for sink strength [67]. Recent studies have proved that high expression of SUS could promote sucrose accumulation [15,68]. The higher expression of present SUS *arahy.DW5X6C* indicated a stronger sink strength in sucrose accumulation in ICG 12625 than in Zhonghua 10. Therefore, it could be concluded that the final high sucrose content in ICG 12625 was a result of synergetic regulation of these SWEETs and key enzymes.

TFs were important regulators in the gene expression that interacts with cis-elements in the promoter of downstream genes and their important effect in sucrose accumulation; they have been identified as MYB [26], AP2/ERF [69], bZIP [70], and WARKY [25,71]. In order to identify which genes/TFs were more responsible for changes in sucrose content during seed development, WGCNA, a widely applied tool for inferring co-expression network modules, was performed, and 90 TFs related to sucrose metabolism were identified. Promoter analysis combined with co-expression analysis revealed that TFs belonging to ERF, BBR-BPC, bZIP, TCP, and MYB mostly regulated the expression of ten genes (Supplementary Table S7). These results could be helpful to fully understand what molecular mechanisms regulate the higher seed sucrose content of ICG 12625 compared with the lower seed sucrose content of Zhonghua 10.

## 4. Materials and Methods

### 4.1. Plant Materials and Sampling

Two peanut varieties, namely, ICG 12625 (high-sucrose) and Zhonghua 10 (low-sucrose), were planted in an experimental field in 2018 at the Oil Crops Research Institute of the Chinese Academy of Agricultural Sciences (OCRI-CAAS), Wuhan, China. We used the same field management during the whole period of growth to eliminate the impact produced by environmental factors. The two varieties had a similar flowering time and maturity period. Following the previous studies [72], seed samples were collected with six biological replicates at 15, 20, 30, 40, 50, 60, and 70 days after flowering (DAF), representing the S1, S2, S3, S4, S5, S6, and S7 stages, respectively (Figure 1A). S1 and S2 were the early-development phase; S3, S4, and S5 were the developing phase; and S6 and S7 were the seed maturation phase. At the 15 DAF, 20 DAF, and 30 DAF stages, at least 30 seeds were harvested for each biological replicate. For other stages, 10 seeds were collected for each biological replicate. The seeds with three biological replicates from each stage were used to evaluate sucrose content, oil content, and protein content. The remaining three biological replicates were used for sequencing and performing RNA-seq analysis.

### 4.2. Determination of Sucrose Content, Oil Content, and Protein Content

The fresh seeds were first treated at 100 °C for 30 min, and then dried at 60 °C to achieve a constant weight. After drying these seeds, sucrose content was quantified using high performance liquid chromatography (HPLC) (following the method described by Dumont E.) [73]. Initially, the dried seeds were crushed by a grinder. One g of power sample (filtered by a 20-mesh sieve) was accurately weighed and dissolved in 10 mL of 80% ethanol (V(ethanol): V(water) = 80:20)) followed by heating at 80 °C in a water bath for 30 min. The resulting mixture was then centrifuged at 25 °C in 12,000 rpm for 10 min. The supernatant solution was filtered through a 0.45 μm Millipore membrane into the 1 mL sample bottle for high performance liquid chromatography with refractive index detection (HPLC-RID) analysis.

The oil content was quantified using the Soxhlet extraction method. Firstly, filter papers with an appropriate size were dried in an oven at 105 °C to a constant weight, then, they were put in a drier and cooled to room temperature. The weight of the filter paper was weighed using an electronic balance (one ten-thousandth) and recorded as M0. The power sample (~0.5 g) and filter paper were weighed together and recorded as M1. After wrapping the power sample with filter paper, it was put into the Soxhlet extractor with 60 mL of petroleum ether (60–90 °C) and soaked for 30 min. The reflux time of the Soxhlet extractor was 3 h, and the temperature was 105 °C. When finished, the filter paper was taken out and dried in an oven with 105 °C until achieving a constant weight. Then, it was put into drier and cooled to room temperature. The weight of the filter paper was weighed by electronic balance (one ten-thousandth) and recorded as M2. The oil content of the seed was calculated according to the following formula:

Oil content(%) = (M1−M2)/(M1−M0) × 100%

Protein analysis was conducted using a Kjeldahl apparatus composed of a Tecator digestion system and a Tecator distillation unit following the steps described by Beljkaš [74]. Samples were weighed (~0.5g) and transferred into a Kjeldahl digestion flask containing 10.0 g of catalyst (prepared by mixing 9 g of K2SO4 and 1 g of CuSO4 × 5H2O) and 25 mL of concentrated H2SO4. After 2.5 h of digestion in the Tecator digestion system and cooling to room temperature, 80 mL of NaOH base (mass fraction w = 33%) was added to each flask. By distillation, ammonium hydroxide was trapped as ammonium borate in a boric acid solution. The total nitrogen was determined by titration with standardized HCl to a mixed indicator endpoint. Then, the total nitrogen content was converted to protein content by using a conversion factor (5.46 for peanut) [75]. The protein content was calculated as the following formula, where c was the conversion factor:

Protein content(%) = N(%) × c

### 4.3. RNA-Seq and Data Processing

The seeds of seven developmental stages with three biological replications in two varieties were immediately frozen in liquid nitrogen for RNA extraction. Total RNA was extracted using TRIzol Reagent (TaKaRa, Inc., Dalian, China) according to its protocol. RNA degradation and contamination were monitored on 1% agarose gels. RNA quality and purity were checked using Agilent 2100 and NanoDrop. All the 42 libraries (14 samples with three biological replicates) were sequenced on the Illumina platform (HiSeq 2000) to generate 150-nucleotide-long paired-end sequence reads. The raw reads from RNA-seq were trimmed using Trimmomatic [76] to remove the adapter and low-quality sequences. The read quality was assessed using FastQC (http://www.bioinformatics.babraham.ac.uk/projects/fastqc/; accessed on 6 June 2020). The program HISAT2 [77] with default parameters was used to align sequencing reads to the cultivated peanut Tifrunner genome (http://www.peanutbase.org; accessed on 8 June 2020). Annotated reference genes were identified using the reference genome GFF3 annotation file (arahy.Tifrunner.gnm2.ann1.4K0L.gene_models_main.gff3). The gene expression was measured by fragments per kilobase of transcript length per million mapped reads (FPKM), and the FPKM for each annotated reference genes were calculated using StringTie (v1.3.4) [78]. With the resulting FPKM data of the expressed genes (mean FPKM > 0), principal components analysis (PCA) and Spearman correlation analysis were used to compare gene expression profiles among all 42 samples by the “prcomp” and “cor.test” functions in R (http://www.R-project.org; accessed on 13 June 2020), respectively. Statistical analysis of differential gene expression was conducted with DESeq2 (v1.18.1) [79]. The genes exhibiting a difference of at least a two-fold change with a corrected *p* value after adjusting with a false discovery rate (*q*-value) <0.05 were considered to be significantly differentially expressed. Venn diagrams, correlation heatmaps, and gene expression heatmaps in this study were created by TBtools [80]. Statistical analysis such as the independent samples *t*-test and Bonferroni’s multiple comparison test was performed by SPSS 25.0 software.

### 4.4. Identification of Stage-Special Expression Genes

The genes specifically expressed in each tissue were identified using a stage specificity (SS) scoring algorithm that compares the expression level of a gene in a given compartment with its maximal expression level in the other compartments [81,82,83]. SS scores range from 0 to 1, and the higher the SS score of a gene for a tissue, the more likely the gene is specifically expressed in that tissue. The SS score ≥0.5 was used to identify the genes specially expressed at a particular stage of development for each variety.

### 4.5. Phylogenetic Analysis of SWEET Genes

The protein sequences of SWEETs in peanut and *Arabidopsis* were downloaded from the database of PeanutBase (http://www.peanutbase.org; accessed on 18 July 2020) and TAIR (https://www.arabidopsis.org; accessed on 18 July 2020), respectively. The phylogenetic trees were constructed based on the neighbor-joining (NJ) method using the parameters referred to in Liu et al. [84].

### 4.6. Gene Ontology Enrichment Analysis and Metabolism Pathway Analysis

Gene ontology (GO) enrichment analysis was performed for stage-special expression genes and DEGs using TBtools [80]. The *p*-value for enrichment was calculated for each represented GO term and corrected via the Benjamini–Hochberg error correction method. Further, pathway enrichment analysis of DEGs was performed using MapMan (v3.6.0R1) [85]. The mapping file for the software MapMan was generated by using the Mercator web application, which took the peanut protein fasta annotation file (arahy.Tifrunner.gnm2.ann1.4K0L.protein.faa) as input.

### 4.7. Gene Co-Expression Analysis by WGCNA

The co-expression network analysis was performed using the WGCNA package in R [86,87]. The DEGs with very low expression (max FPKM in all samples <2) were not considered for this analysis to avoid inclusion of spurious edges in the networks. Using the FPKM values of the remaining genes, a matrix of pairwise Spearman correlation coefficients (SCCs) between all pairs of genes was generated and transformed into an adjacency matrix (a matrix of connection strengths) using the formula: connection strength (adjacency value) = |(1 + correlation)/2|β. The resulting adjacency matrix was converted to a topological overlap (TO) matrix via the TOM similarity algorithm. WGCNA-unsigned network construction and module detection was conducted using an unsigned type of topological overlap matrix (TOM), a minimal module size of 30, and a branch merge cut height of 0.20. A β value of 7 was selected based on the scale-free topology criterion described by Zhang and Horvath [87]. In the weighted gene co-expression network, the topology overlap measure provided in WGCNA was used to determine edge weight, which reflected the strength of communication between the two genes. The networks were visualized using Cytoscape (v.3.7.2) [88].

### 4.8. Regulation between TF and Genes Related to Sucrose Content

A protein dataset (arahy.Tifrunner.gnm2.ann1.4K0L.protein.faa) was downloaded from the PeanutBase database (https://www.peanutbase.org/; accessed on 16 August 2020) and was used to identify the TFs of *A. hypogaea* according to PlantTFDB v5.0 [89,90]. To analyze TF binding sites upstream of the genes in the regulation network of sucrose metabolism, two kb of the promoter sequences of genes were obtained by Tbtools [80], and these sequences were used to predict the TF binding sites by aligning to *Arabidopsis thaliana* on PlantRegMap [91].

### 4.9. qRT-PCR Validation for DEGs

DEGs were selected for verification by qRT-PCR. Their information of cDNA was retrieved from PeanutBase (https://www.peanutbase.org; accessed on 16 August 2020). Primers specific to the selected genes were designed using Primer Premier 5.0. For each sample, 1 μg of total RNA was converted into cDNA using Thermo Scientific™ RevertAID™ First Strand cDNA Synthesis Kit (ThermoFisher, Wuhan, China) and was subsequently diluted two times with sterile water. The ChamQ Universal SYBR Green qPCR Master Mix (Vazyme Biotech, Wuhan, China) system was used, with *TUA* [92] and *ADH* [93] as the reference genes. Three biological replicates were processed for qRT-PCR, which was performed using a 20-μL reaction mixture containing 1 μL cDNAs, 0.4 μL of each primer (10 μΜ/L), 8.2 μL sterile water, and 10 μL of ChamQ Universal SYBR Green qPCR Master Mix. All reactions were run as duplicates on 96-well plates. PCR reactions were performed under the following conditions: preincubation at 95 °C for 30 s and 39 cycles of 95 °C for 10 s and 60 °C for 30 s. The relative expression levels were calculated using the 2−ΔΔCt method [94] and normalized by the geometric mean of the two stably expressed housekeeping genes [95] for each sample.

### 4.10. Expression Patterns of 10 Genes in RIL Population

In the previous study [38], the transcriptome data of 100 RILs deriving from Zhonghua 10 and ICG 12625 at the S3 stage were available. To identify the DEGs related to sucrose metabolism, 24 RILs including 12 high sucrose content RILs (high group) and 12 low sucrose content RILs (low group) were selected. The difference analysis of gene expression between the two groups was performed by the independent samples *t*-test. The two groups exhibiting a difference with a *p* value < 0.05 were considered to be significantly different.

## Data Availability

The raw sequencing data of the 42 samples have been submitted to the NCBI Sequence Read Archive (SRA, http://www.ncbi.nlm.nih.gov/sra; accessed on 2 February 2021). The BioProject accession number is PRJNA700804. These data can also be obtained from this website: https://www.ncbi.nlm.nih.gov/sra/PRJNA700804; accessed on 16 February 2021.

## Supplementary Materials

The following are available online at https://www.mdpi.com/article/10.3390/ijms22147266/s1.
