# Supplementary material for: Key Regulators of Sucrose Metabolism Identified through Comprehensive Comparative Transcriptome Analysis in Peanuts
Source: Int J Mol Sci. 2021 Jul 6;22(14):7266. doi: 10.3390/ijms22147266 (PMC8306169; doi:10.3390/ijms22147266)
Supplement: Supplementary file 1 [file ijms-22-07266-s001.zip › ijms-1247925-supplementary/Supporting Information/Supporting Information Legends.pdf]

Supporting Information Legends

Supplementary Figure S1 Heatmaps showing correlation between transcriptomes of three biological replicates of each tissue sample from ICG 12625 and Zhonghua 10. The value was Spearman correlation coefficient among the replicates of ICG 12625 (A) and Zhonghua 10 (B). ICG, ICG 12625; ZH, Zhonghua 10; S1-S7, seed development stages; a-c, three biological replicates of each sample.

Supplementary Figure S2 Principal component analysis plot showing clustering of transcriptomes of different stages of seed development in ICG 12625 (ICG_S1-ICG_S7) and Zhonghua 10 (ZH_S1- ZH_S7)

Supplementary Figure S3 GO analysis of stage-special expressed genes at seven stages of seed development. The abscissa represented the number of enriched genes.

Supplementary Figure S4 The expression profiles of all expressed genes related to sucrose metabolism in ICG 12625 and Zhonghua 10. The expression patterns of enzyme genes involved in sucrose metabolism in ICG 12625 (A) and Zhonghua10 (B) as well as SWEET genes (C for ICG 12625 and D for Zhonghua10) were shown. Color scale represented Z-score.

Supplementary Figure S5 The up- and down-regulated DEGs in ICG 12625. (A) Total number of up- and down-regulated DEGs in ICG 12625. (B) The intersection of up- and down-regulated DEGs in ICG 12625 at S1-S7 stage of seed development (The number of genes less than 50 is not given). The strip at the bottom left shows the number of genes included in each DEGs types. The dot and line at the bottom right represent the subsets of DEGs. The DEGs types corresponding to dots were contained in the subtype. The number of relevant genes in each subset is represented in the histogram, which is the upper part of the whole plot.

Supplementary Figure S6 qRT-PCR verification of 14 DEGs with *TUA* and *ADH* as internal reference genes. (A) The y-axis showed the relative gene expression levels analyzed with qRT-PCR and RNA-Seq. The relative expression of each gene was normalized by geometric mean of two stably expressed reference genes. The green columns corresponded with expression data of qRT-PCR, while orange lines denoted data of RNA-Seq (in all cases, means of three repeats). Error

bars represented standard errors (n = 3). (B) Comparison of gene expression ratios from qRT-PCR and RNA-Seq data.

Supplementary Figure S7 Phylogenetic tree representing the relationships of expressed SWEET genes in ICG 12625 and *Arabidopsis*.

Supplementary Figure S8 Heatmap showing the expression levels of DEGs (enzyme genes and SWEET genes) involved in sucrose metabolism in ICG 12625 and Zhonghua 10. Color scale represented log2(FPKM+1). The expression profile of enzyme genes (A and C) and SWEET genes (B and D) in ICG 12625 as well as Zhonghua 10 was shown.

Supplementary Figure S9 The changes of protein content (A), oil content (B) and proportion of sucrose : protein : oil (C) in the seeds of two peanut varieties at different developmental stages (S3-S7). The value was an average of three biological replications at each stage and the error bar indicated standard error. * and ** represent significant ($P < 0.05$) and extremely significant ($P<0.01$) differences between the two varieties. Different capital or small letters indicate that the oil or protein in the seeds of a variety was significant different ($P < 0.05$).

Supplementary Figure S10 The ratios of oil, protein and sucrose during seed development (S3-S7). The oil/sucrose, protein/sucrose and oil/protein were shown in (A), (B) and (C), respectively.

Supplementary Figure S11 Metabolic pathways analysis of DEGs at S1, S4 and S5 stage. The DEGs at S1, S4 and S5 stage (|fold change| ≥2, q-value ≤0.05) between ICG 12625 and Zhonghua 10 were loaded into MapMan to generate the overview. Red and blue colors represent upregulated and downregulated genes in ICG 12625 as compared with Zhonghua 10, respectively.

Supplementary Table S1 Sucrose content of ICG 12625 and Zhonghua 10 during seed development.

Supplementary Table S2 Summary of read data generated, quality control and mapping rates on the peanut genome for different samples in ICG 12625 and Zhonghua 10.

Supplementary Table S3 The detailed information of stage-special expressed genes involved in sucrose metabolism.

Supplementary Table S4 The number of DEGs involved in different metabolic pathways based on MapMan analysis.

Supplementary Table S5 The information of primers for real-time quantitative PCR.

Supplementary Table S6 The detailed information of DEGs involved in sucrose metabolism.

Supplementary Table S7 Number of TF-binding sites on the promoter (2kb upstream) of genes using PlantRegMAP.
