# Supplementary figures and images for: Key Regulators of Sucrose Metabolism Identified through Comprehensive Comparative Transcriptome Analysis in Peanuts
Source: Int J Mol Sci. 2021 Jul 6;22(14):7266. doi: 10.3390/ijms22147266 (PMC8306169; doi:10.3390/ijms22147266)

A

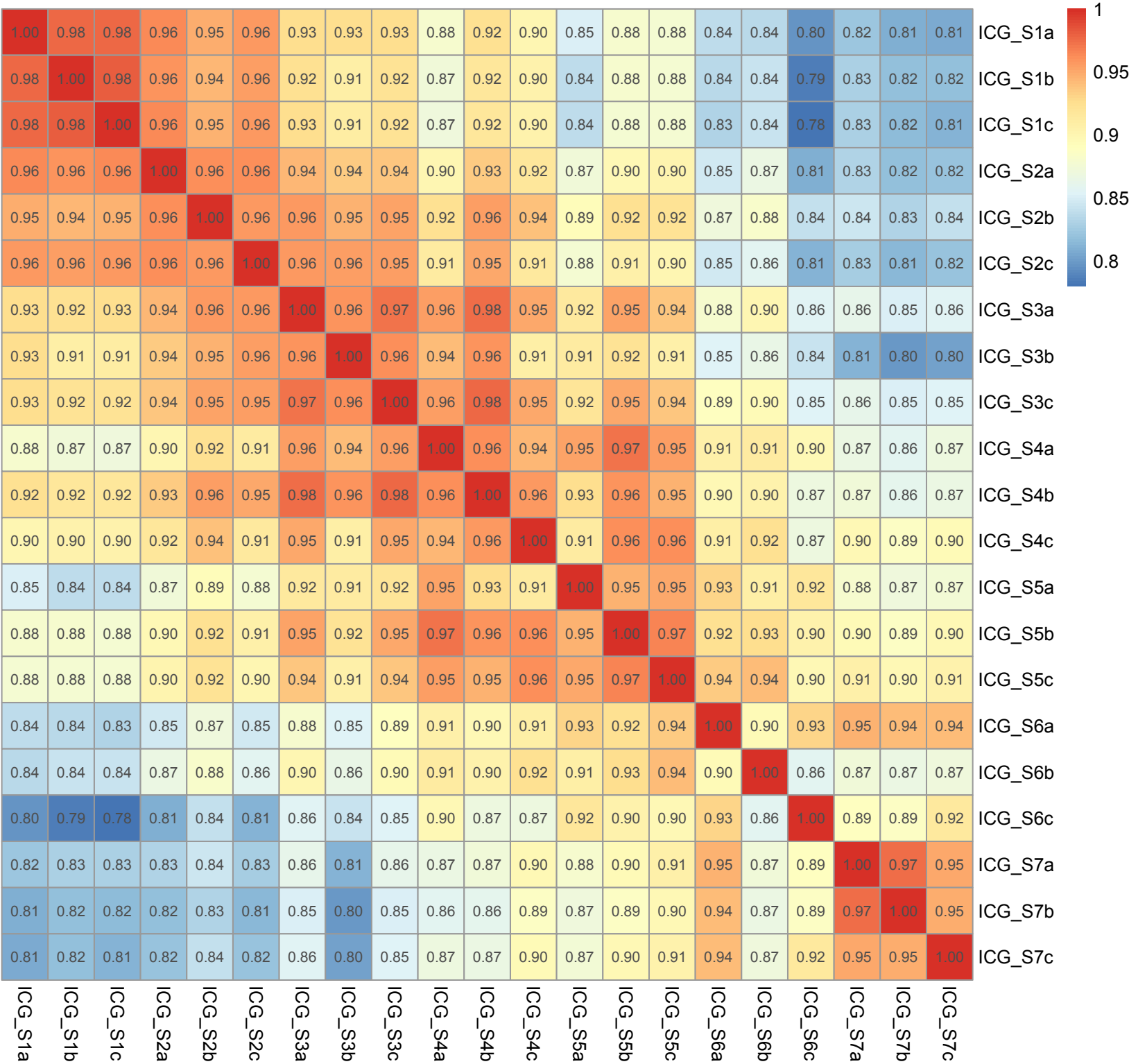

B

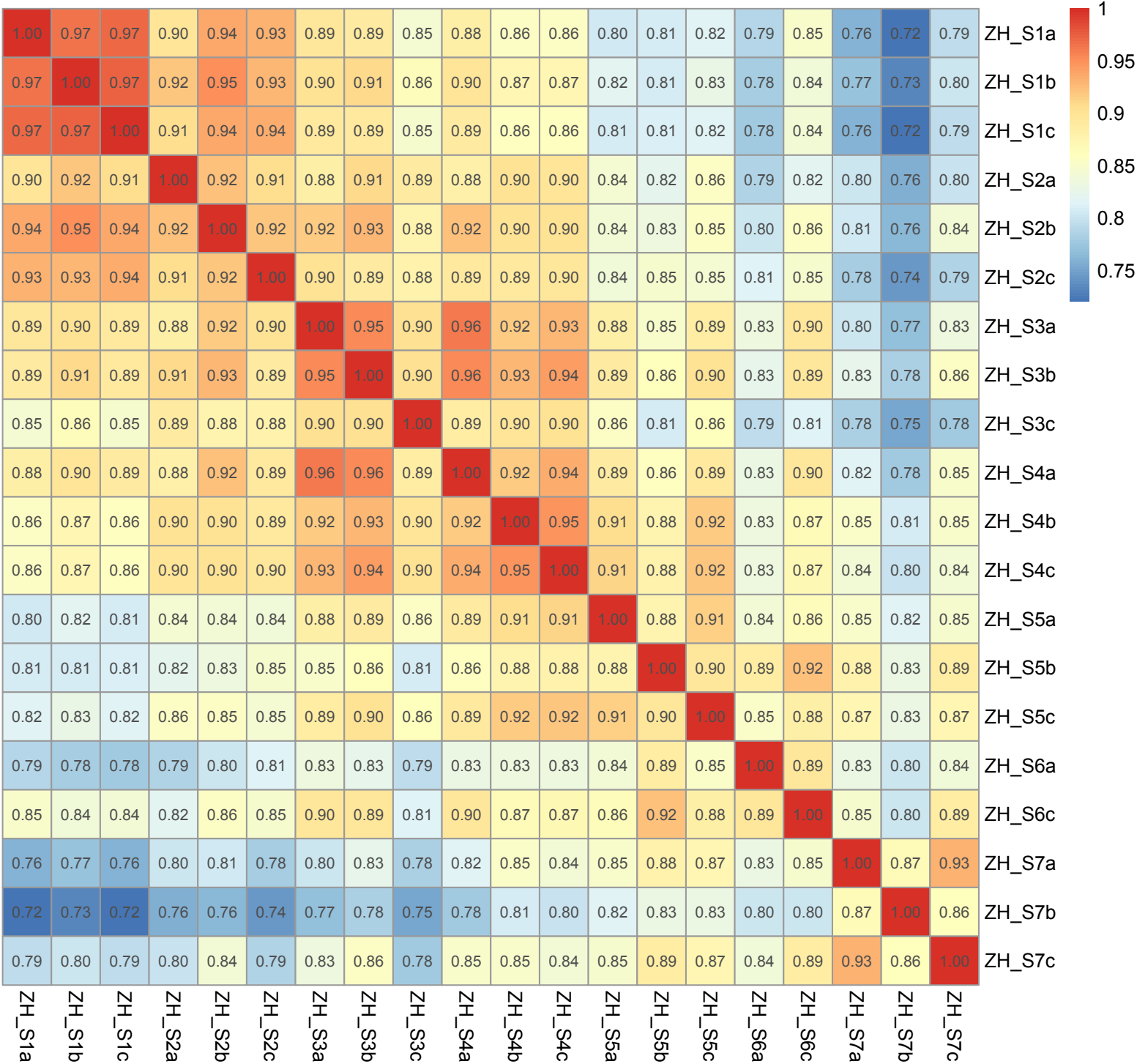

Supplement: Supplementary file 1 [file ijms-22-07266-s001.zip › ijms-1247925-supplementary/Supporting Information/Supplementary Figure S1.pdf]

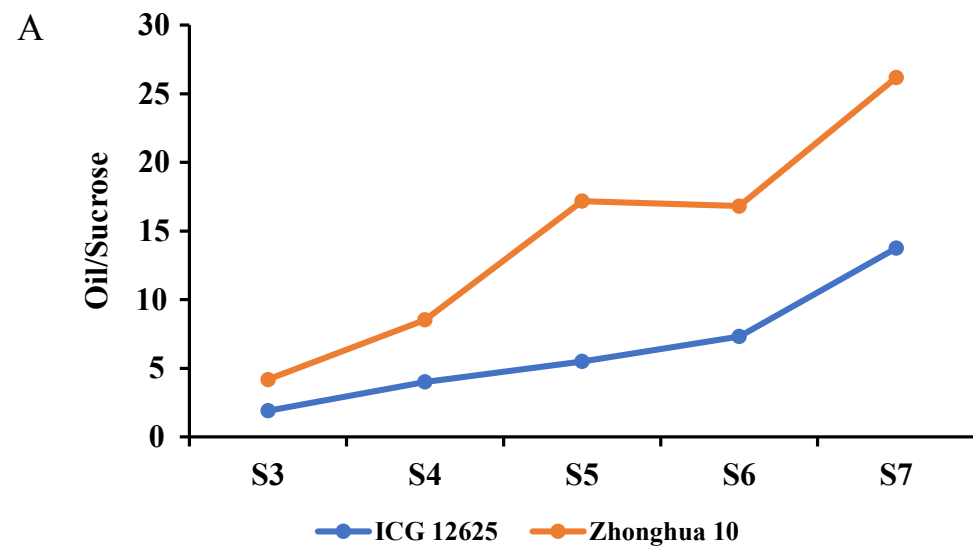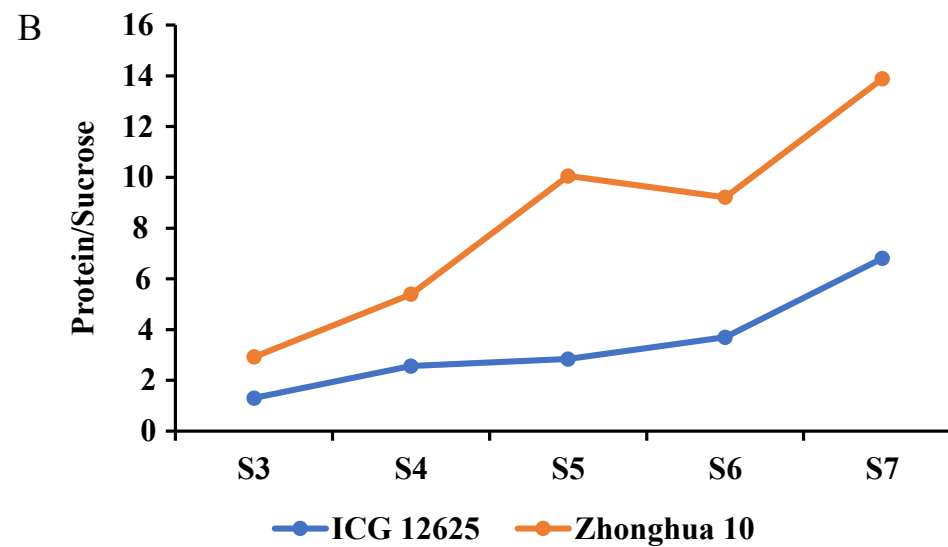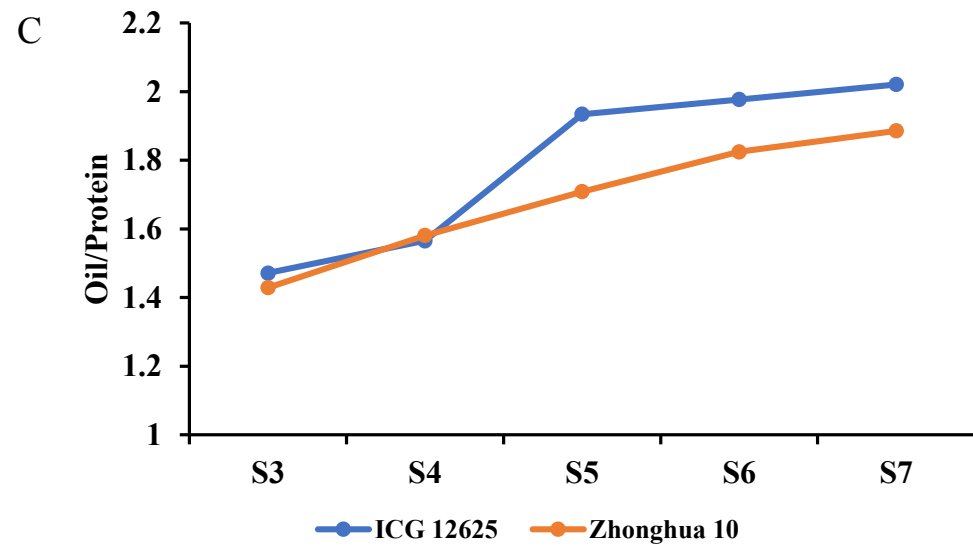

Supplement: Supplementary file 1 [file ijms-22-07266-s001.zip › ijms-1247925-supplementary/Supporting Information/Supplementary Figure S10.pdf]

# PCA

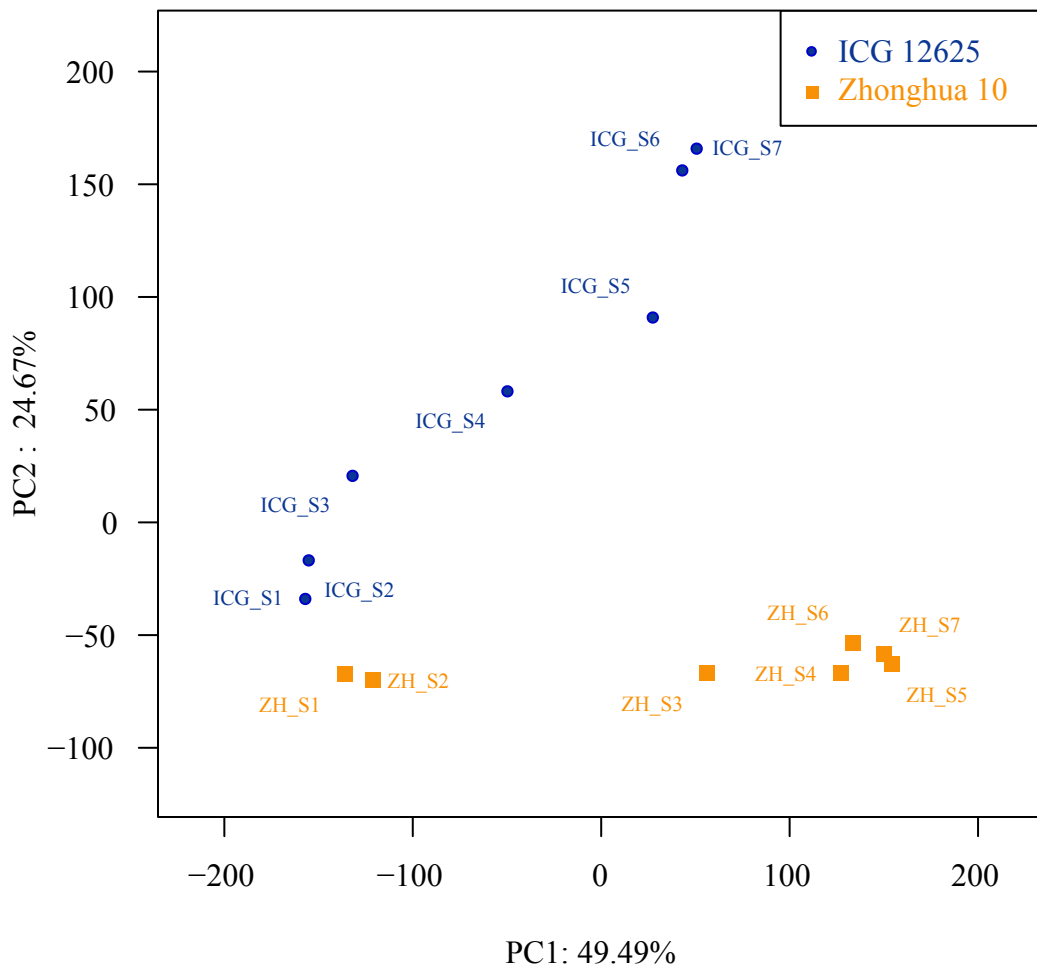

Supplement: Supplementary file 1 [file ijms-22-07266-s001.zip › ijms-1247925-supplementary/Supporting Information/Supplementary Figure S2.pdf]

S1

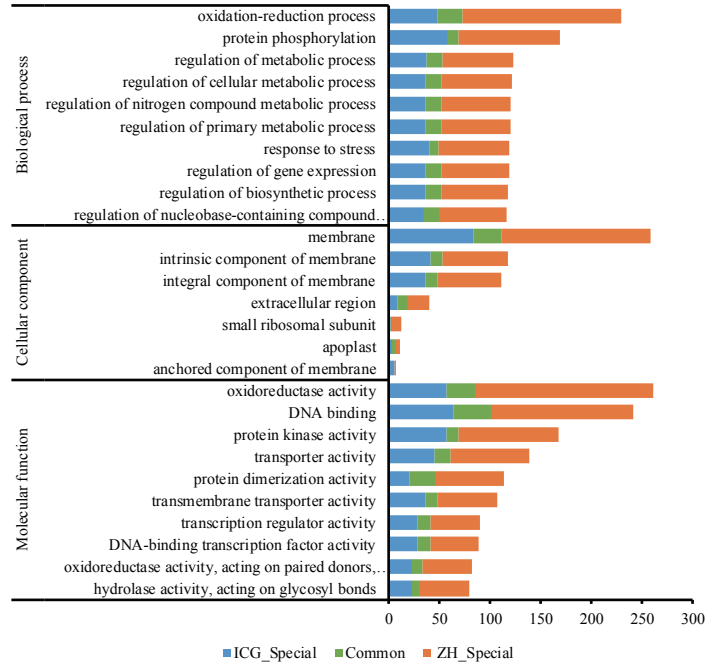

S2

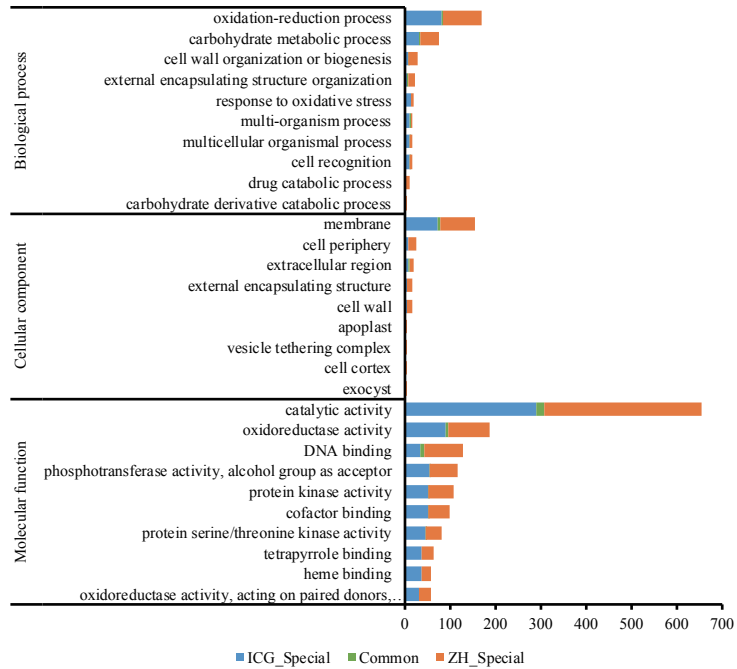

S3

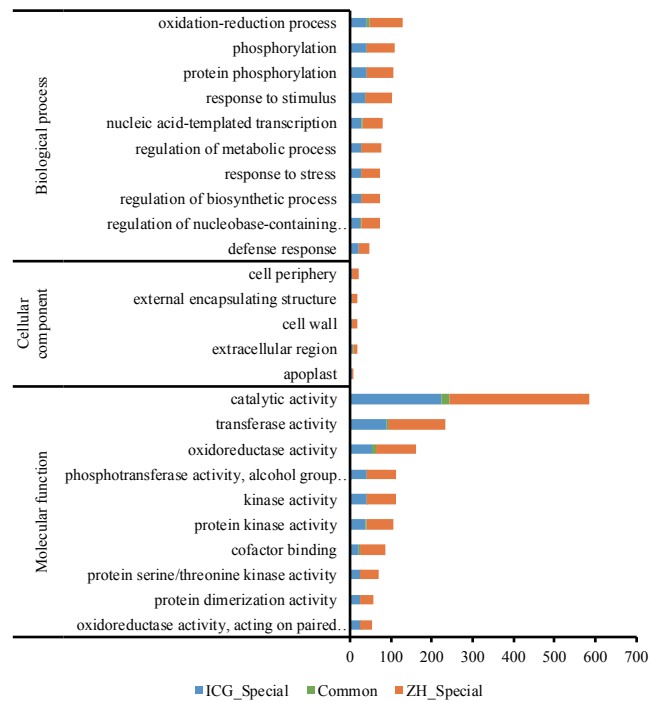

S4

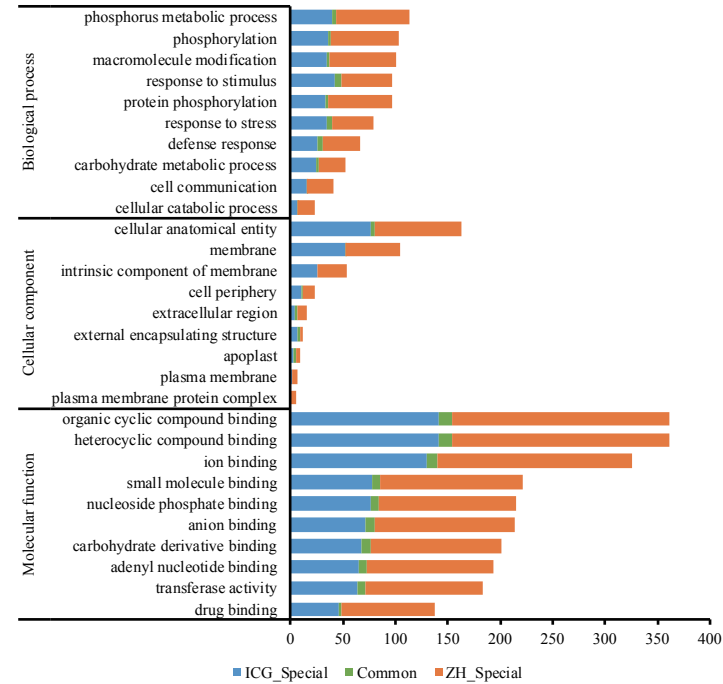

S5

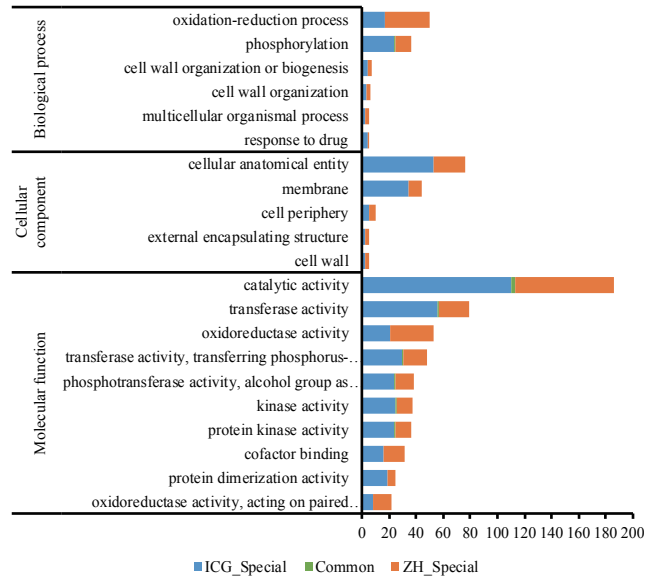

S6

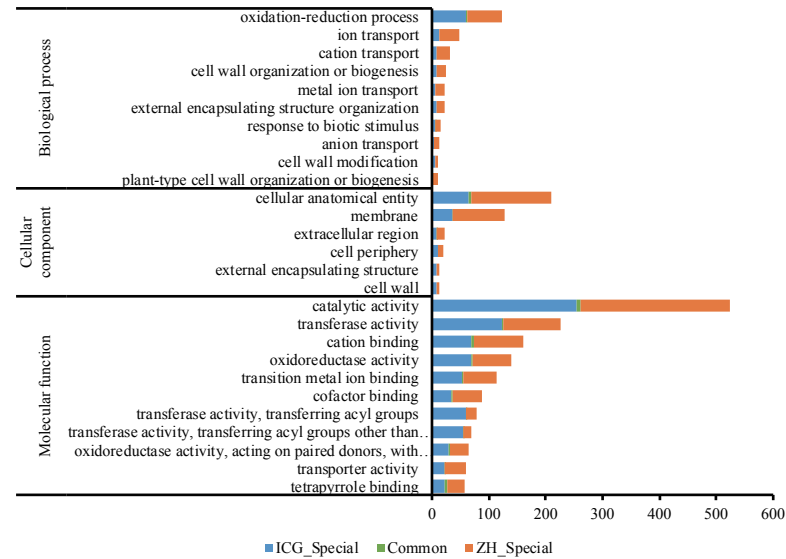

S7

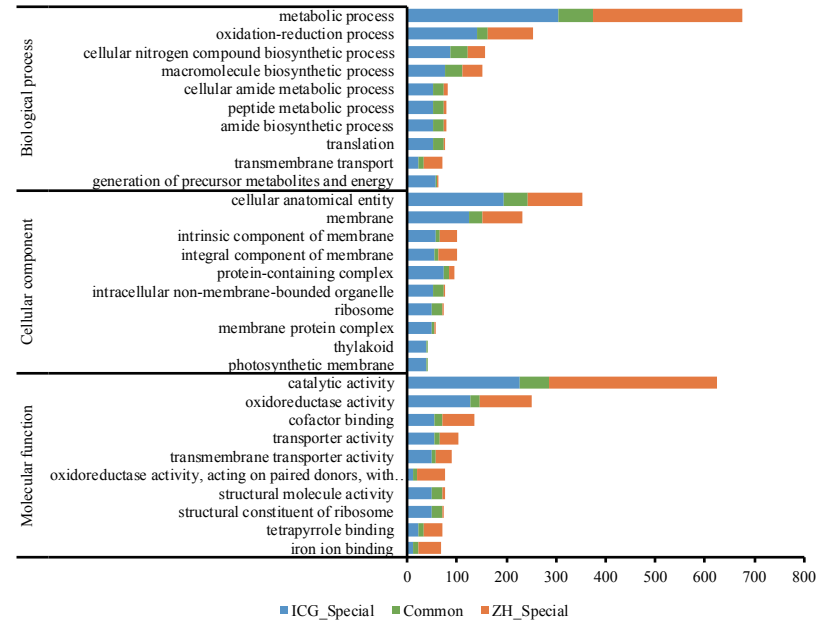

Supplement: Supplementary file 1 [file ijms-22-07266-s001.zip › ijms-1247925-supplementary/Supporting Information/Supplementary Figure S3.pdf]

A

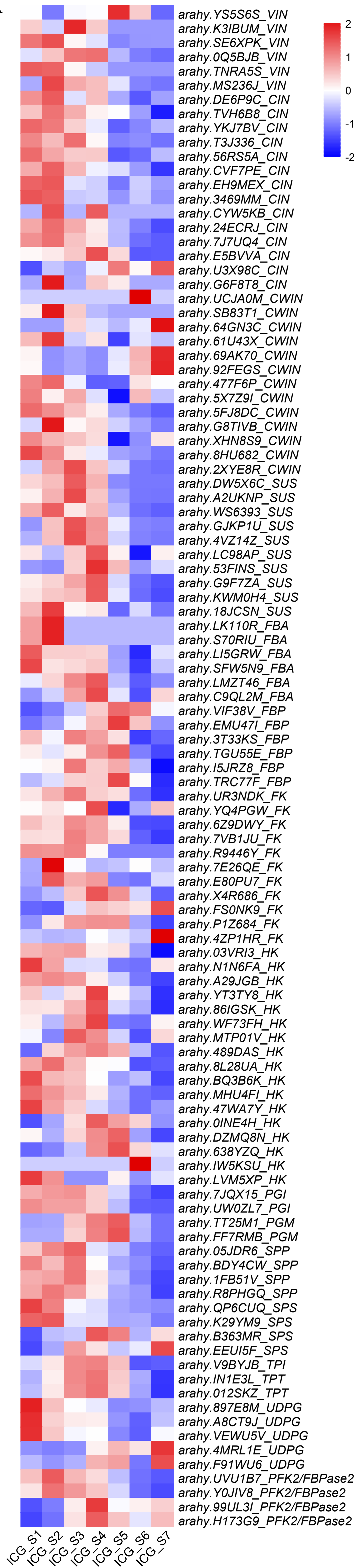

B

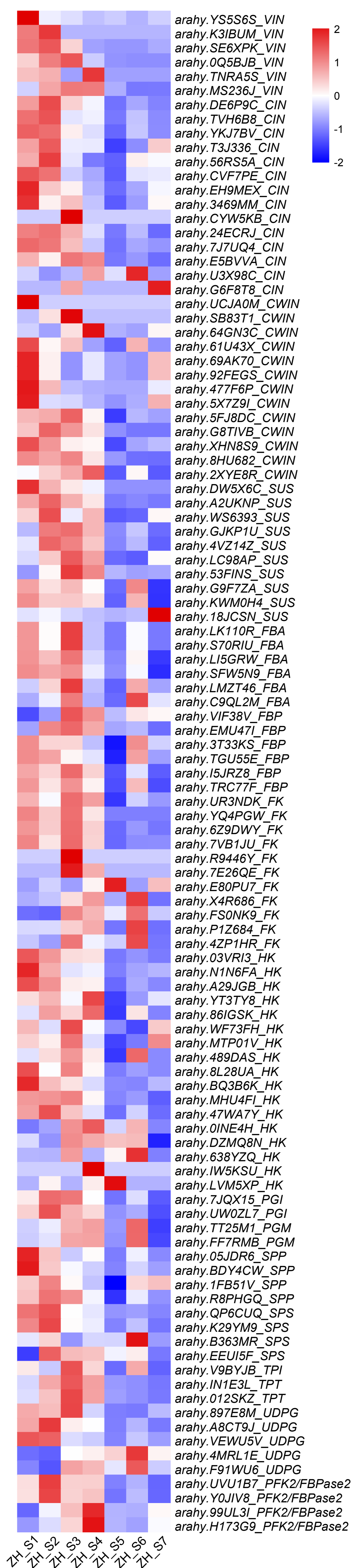

C

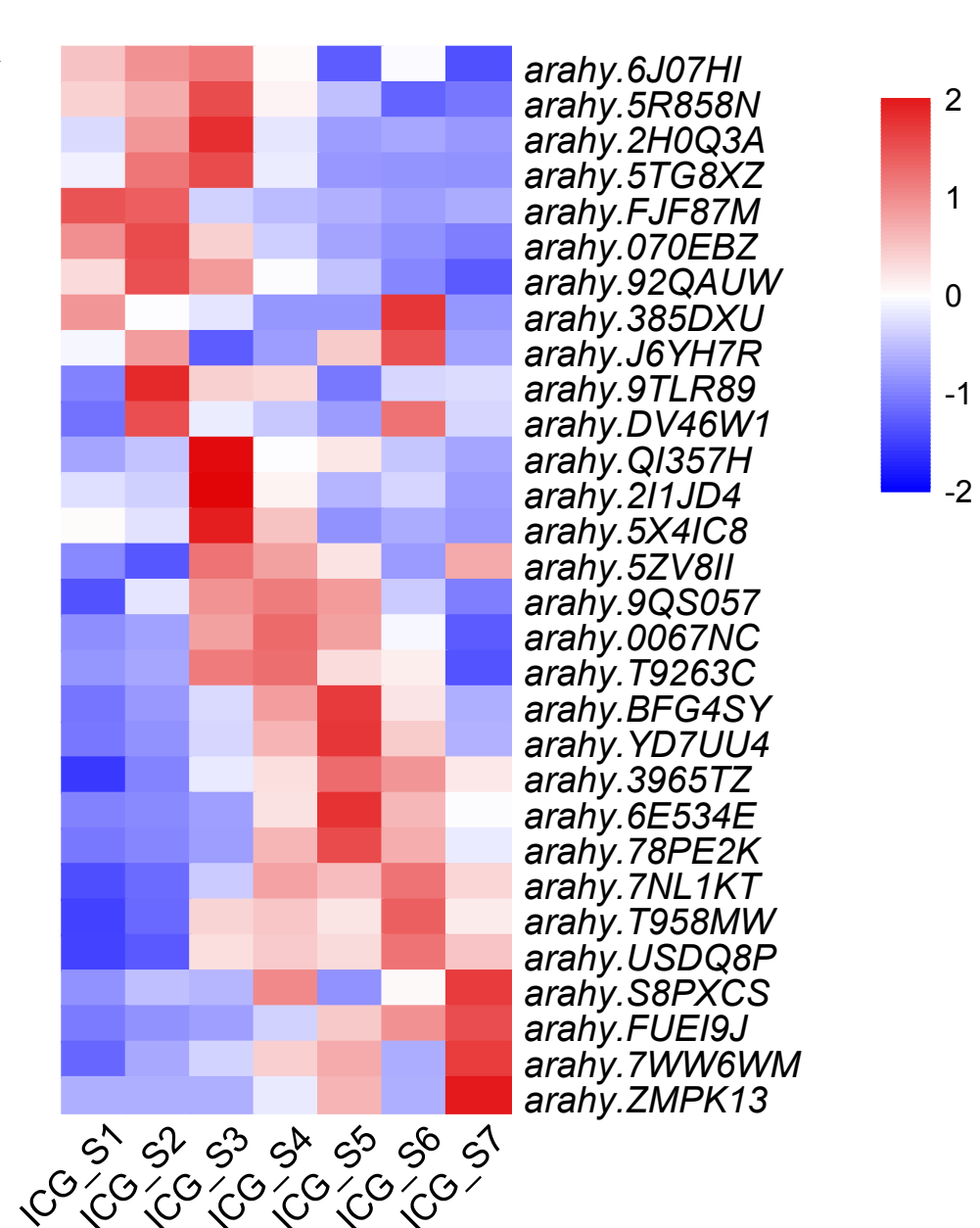

D

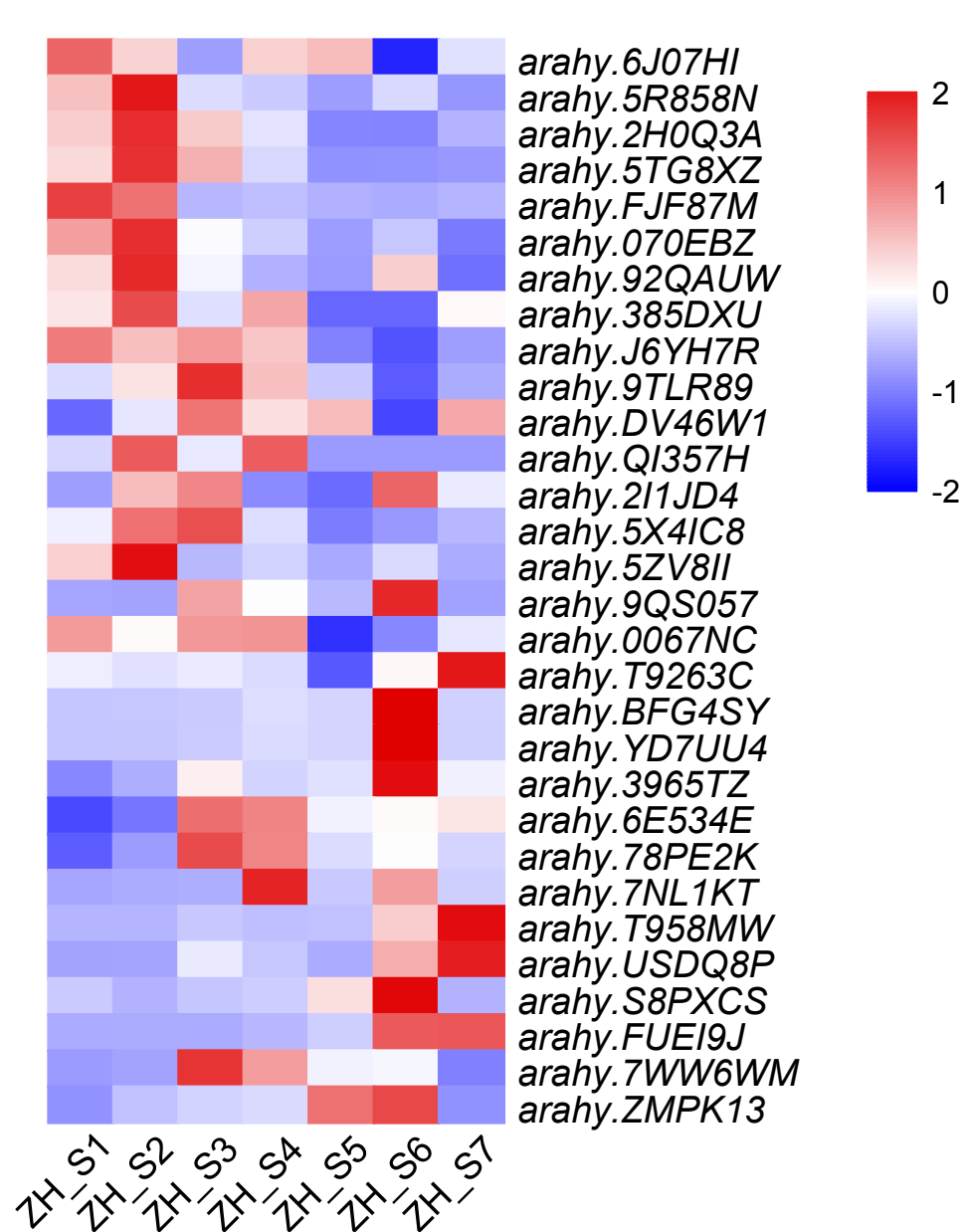

Supplement: Supplementary file 1 [file ijms-22-07266-s001.zip › ijms-1247925-supplementary/Supporting Information/Supplementary Figure S4.pdf]

A

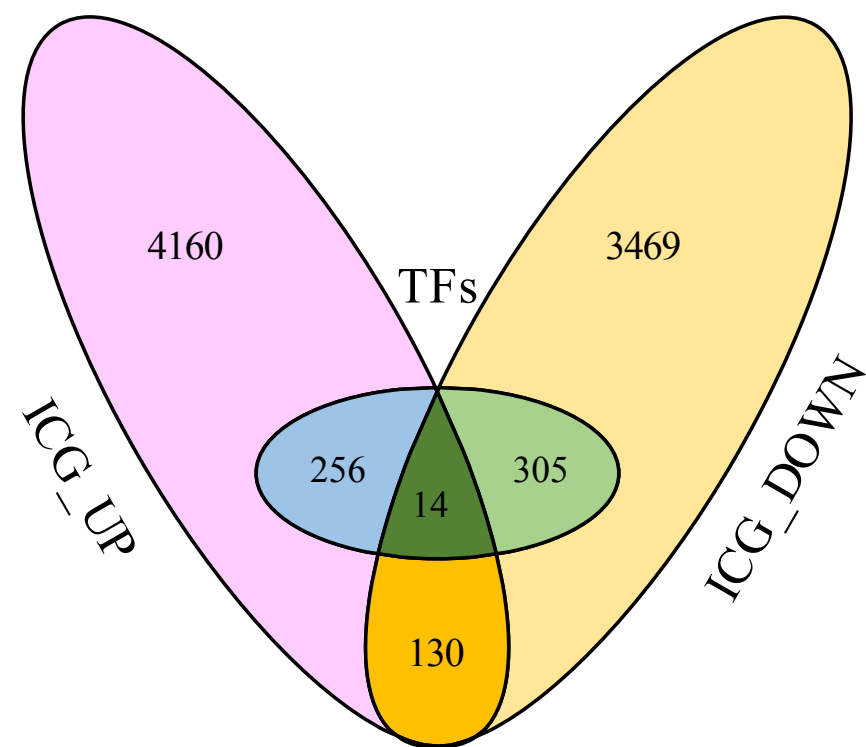

B

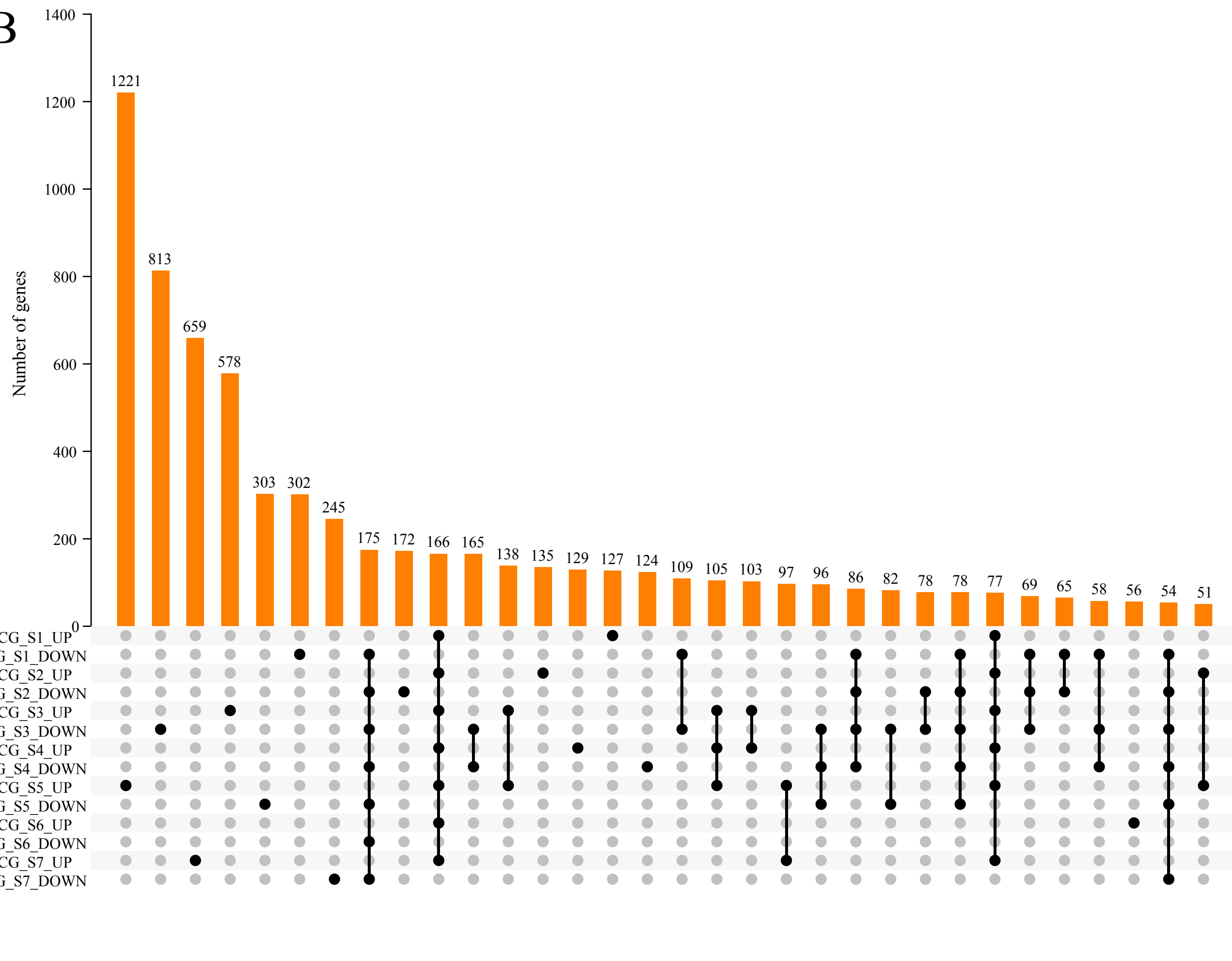

Supplement: Supplementary file 1 [file ijms-22-07266-s001.zip › ijms-1247925-supplementary/Supporting Information/Supplementary Figure S5.pdf]

A

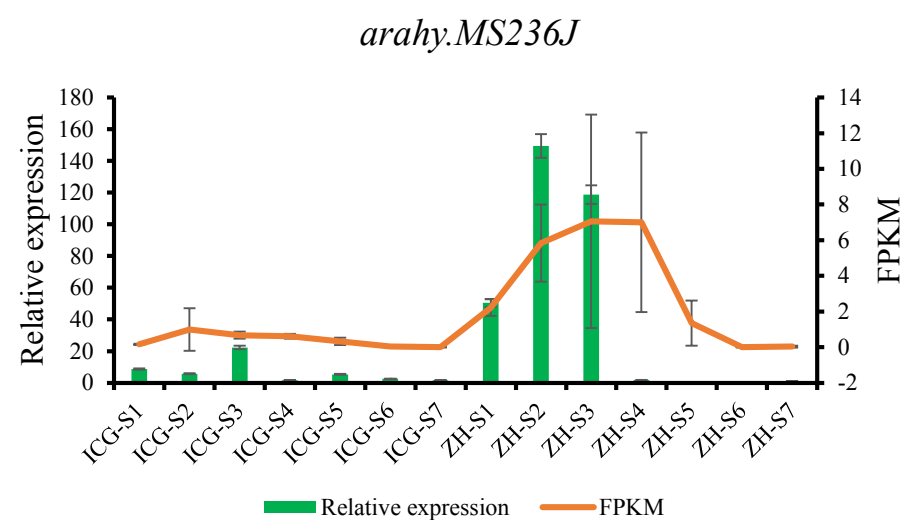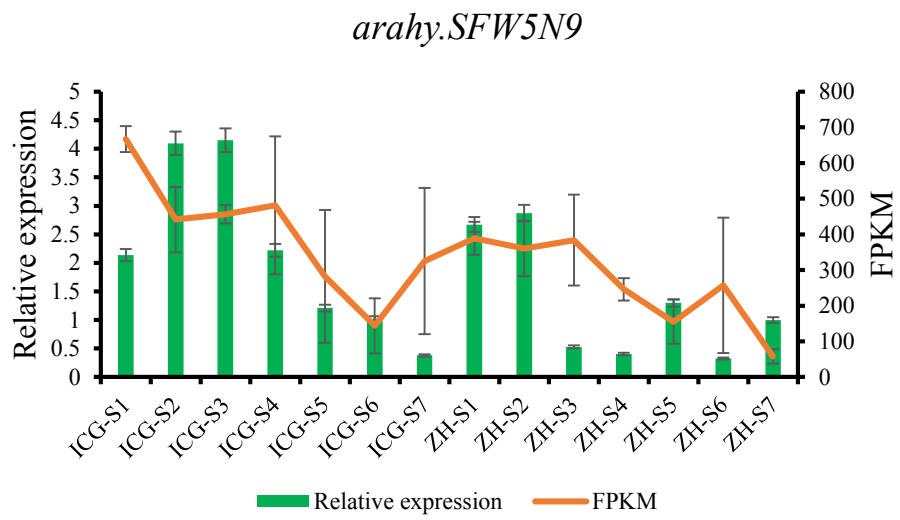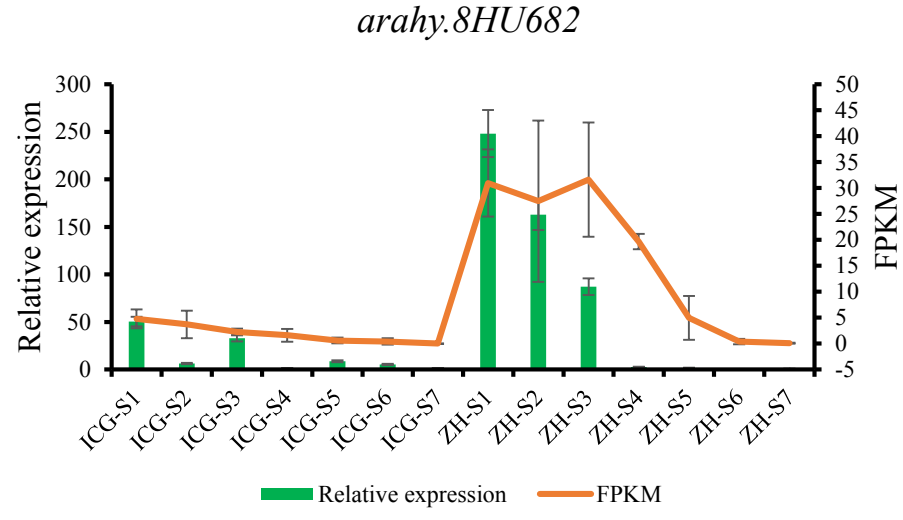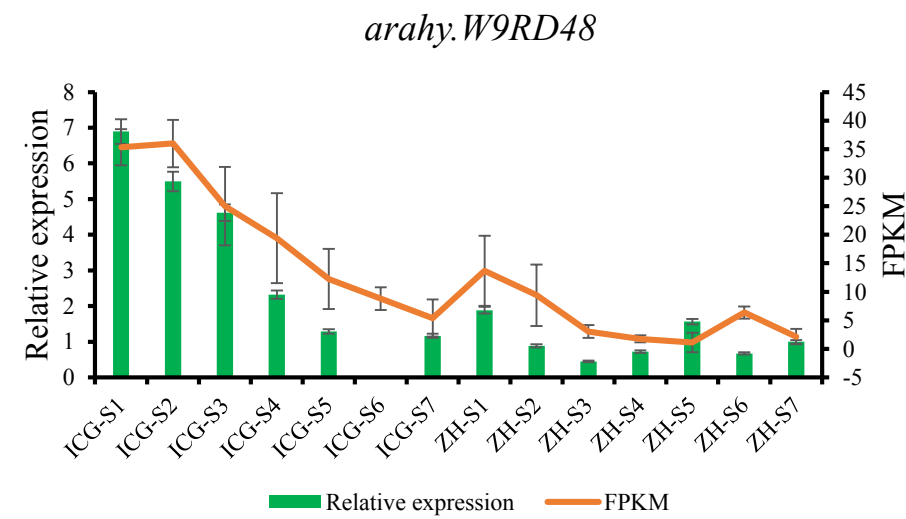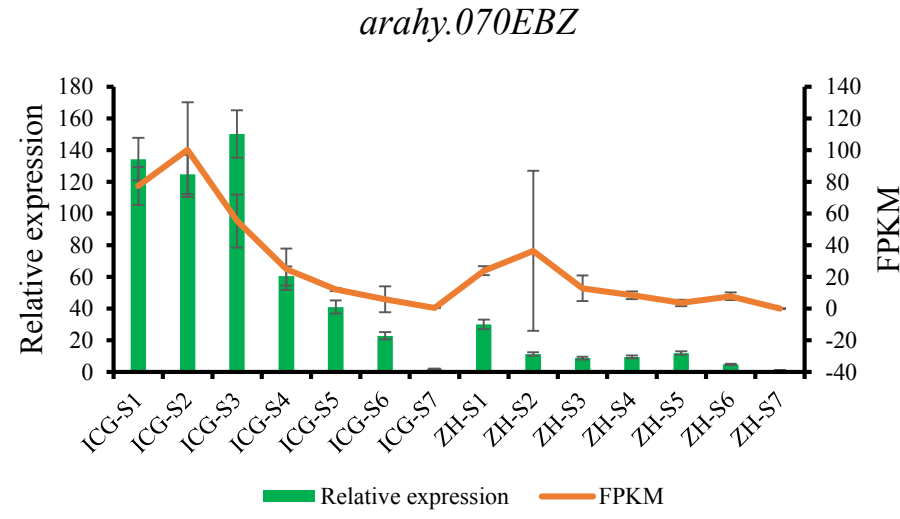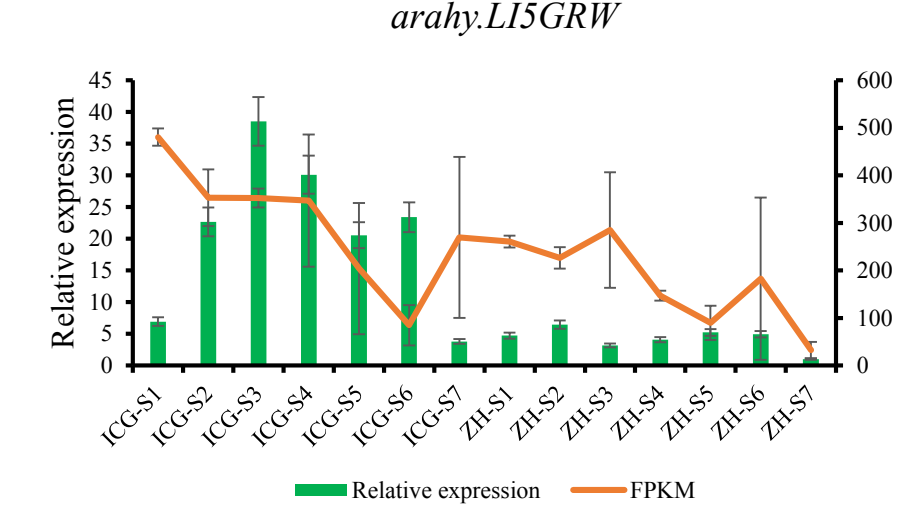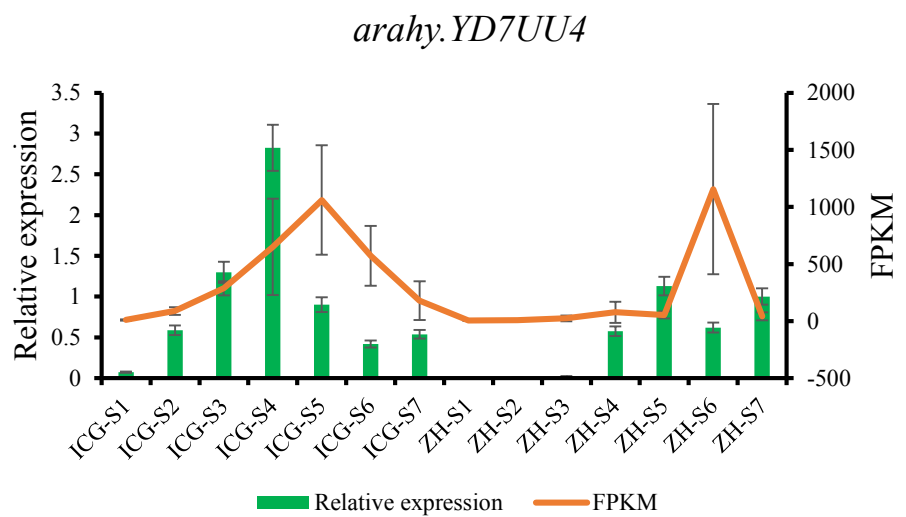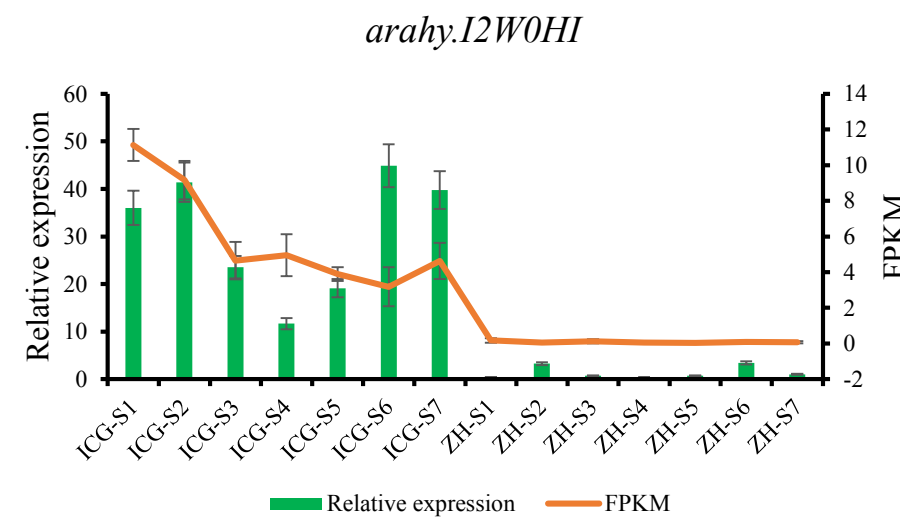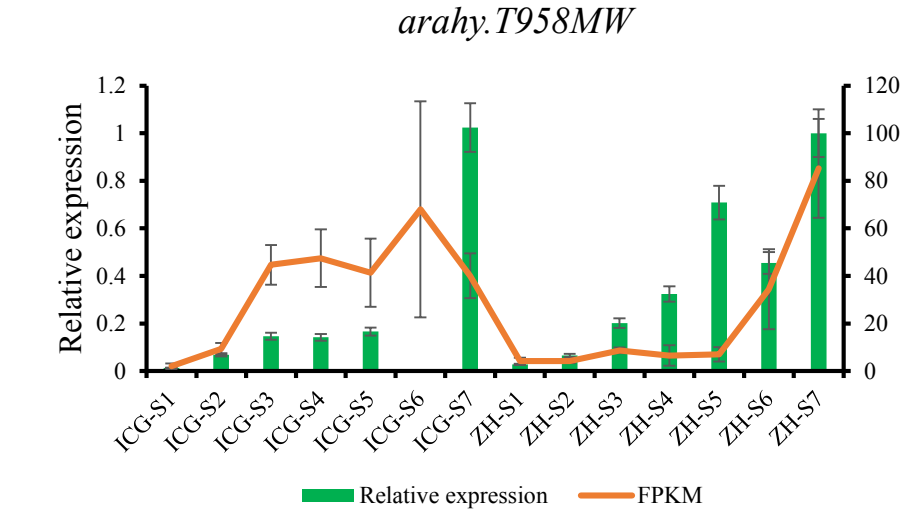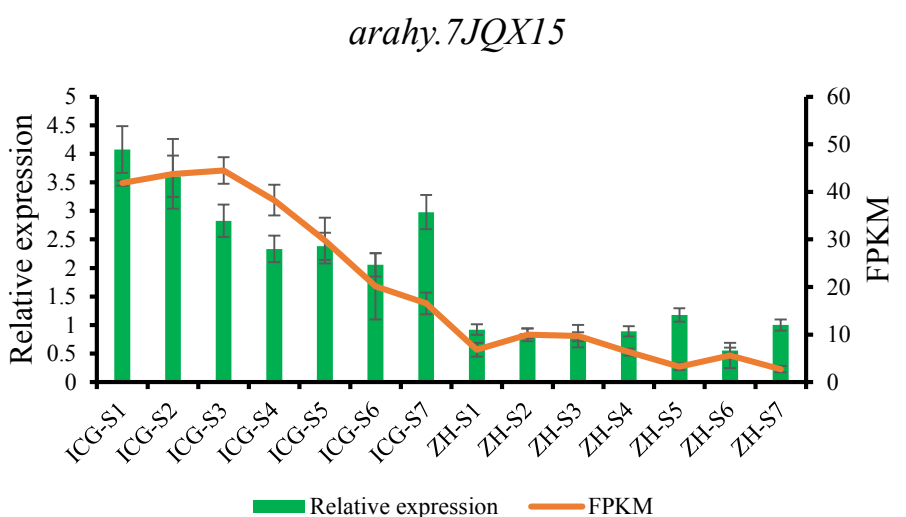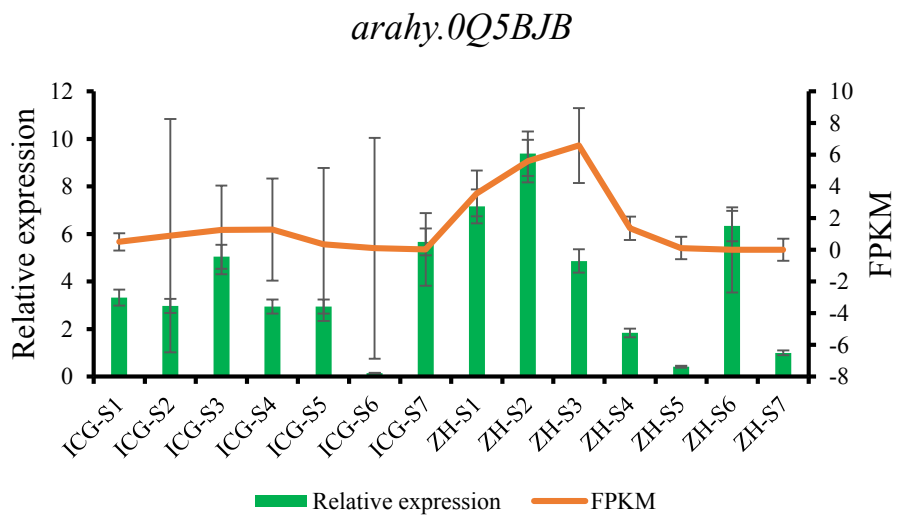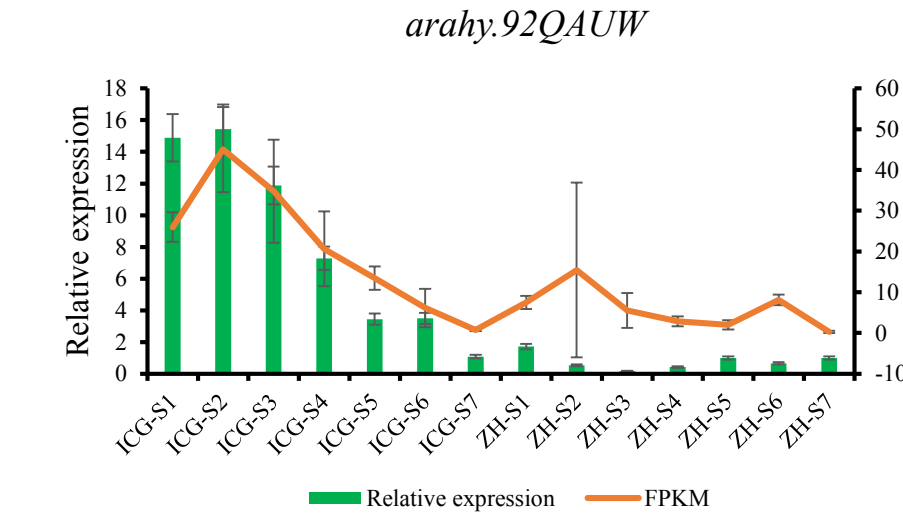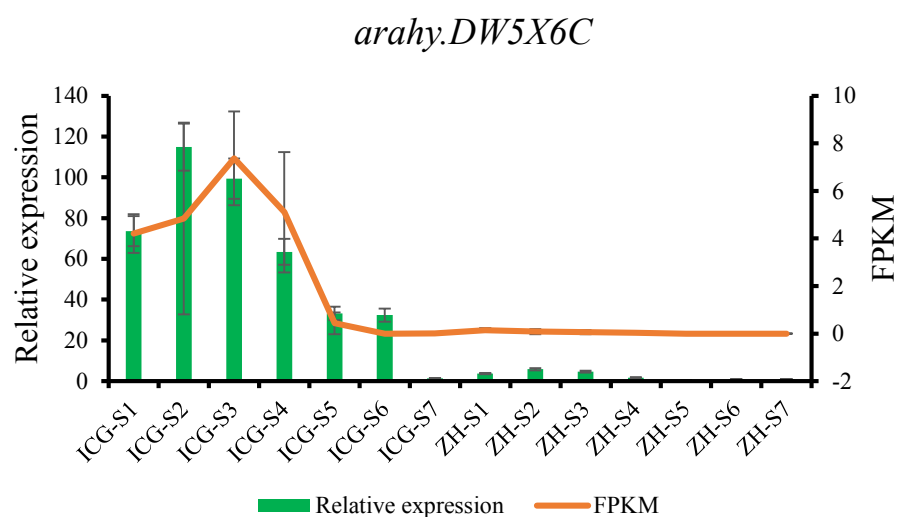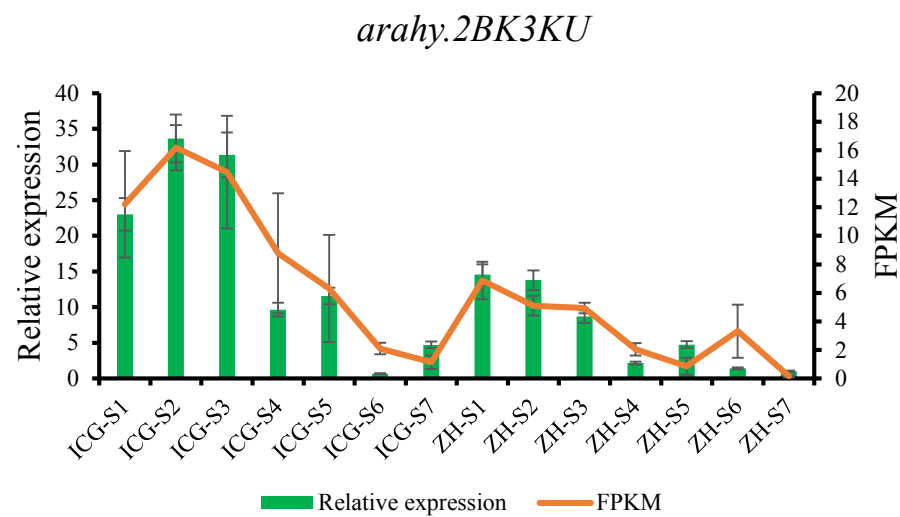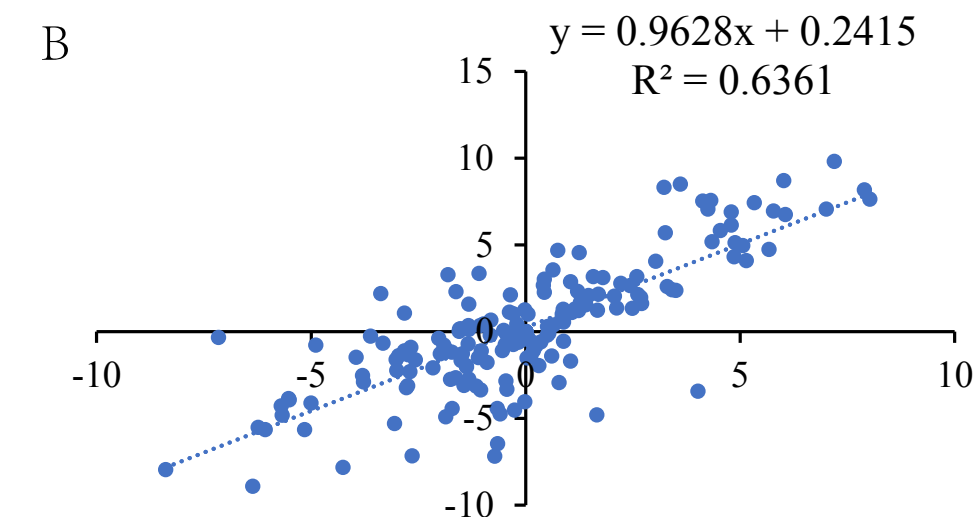

Supplement: Supplementary file 1 [file ijms-22-07266-s001.zip › ijms-1247925-supplementary/Supporting Information/Supplementary Figure S6.pdf]

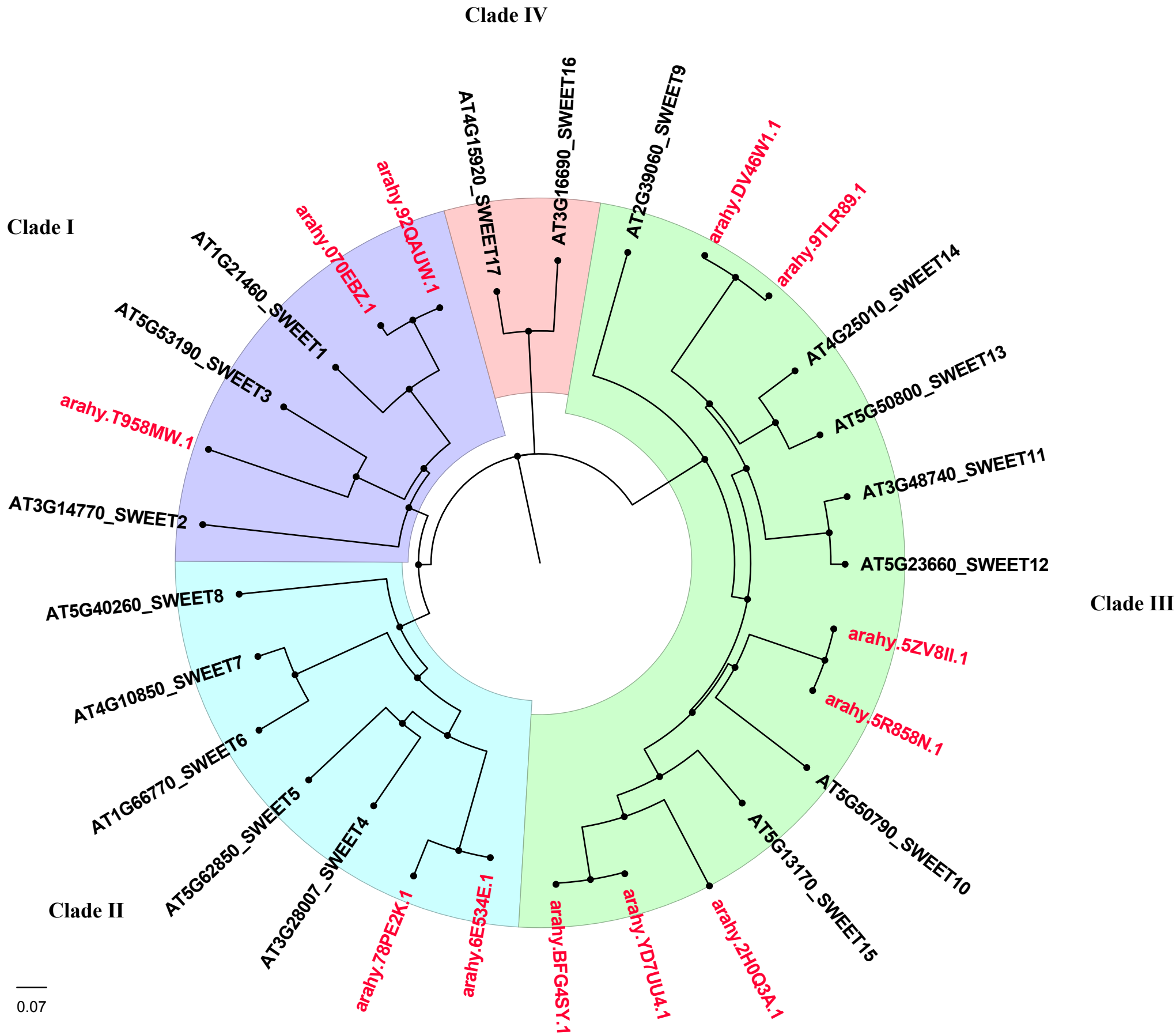

Supplement: Supplementary file 1 [file ijms-22-07266-s001.zip › ijms-1247925-supplementary/Supporting Information/Supplementary Figure S7.pdf]

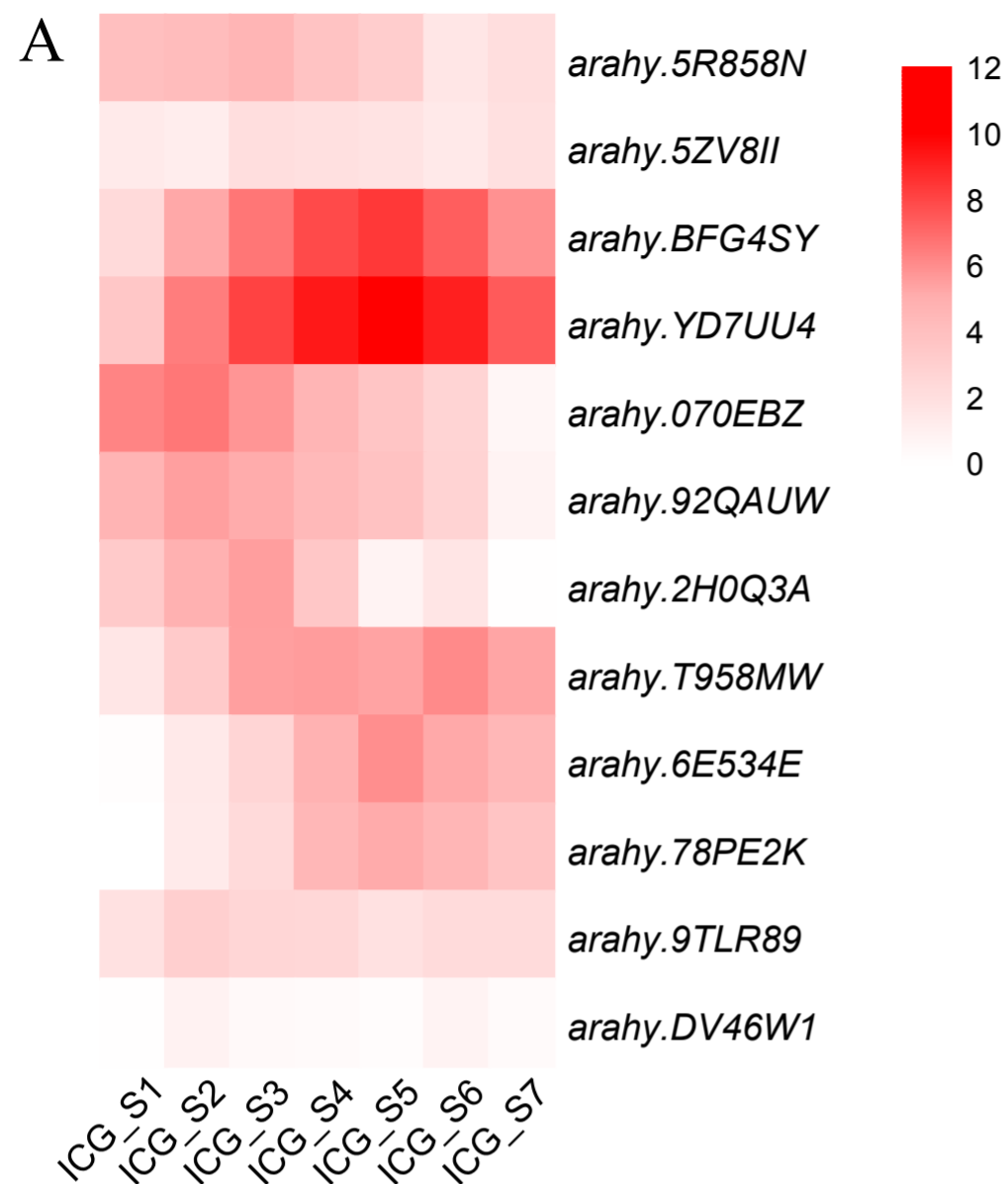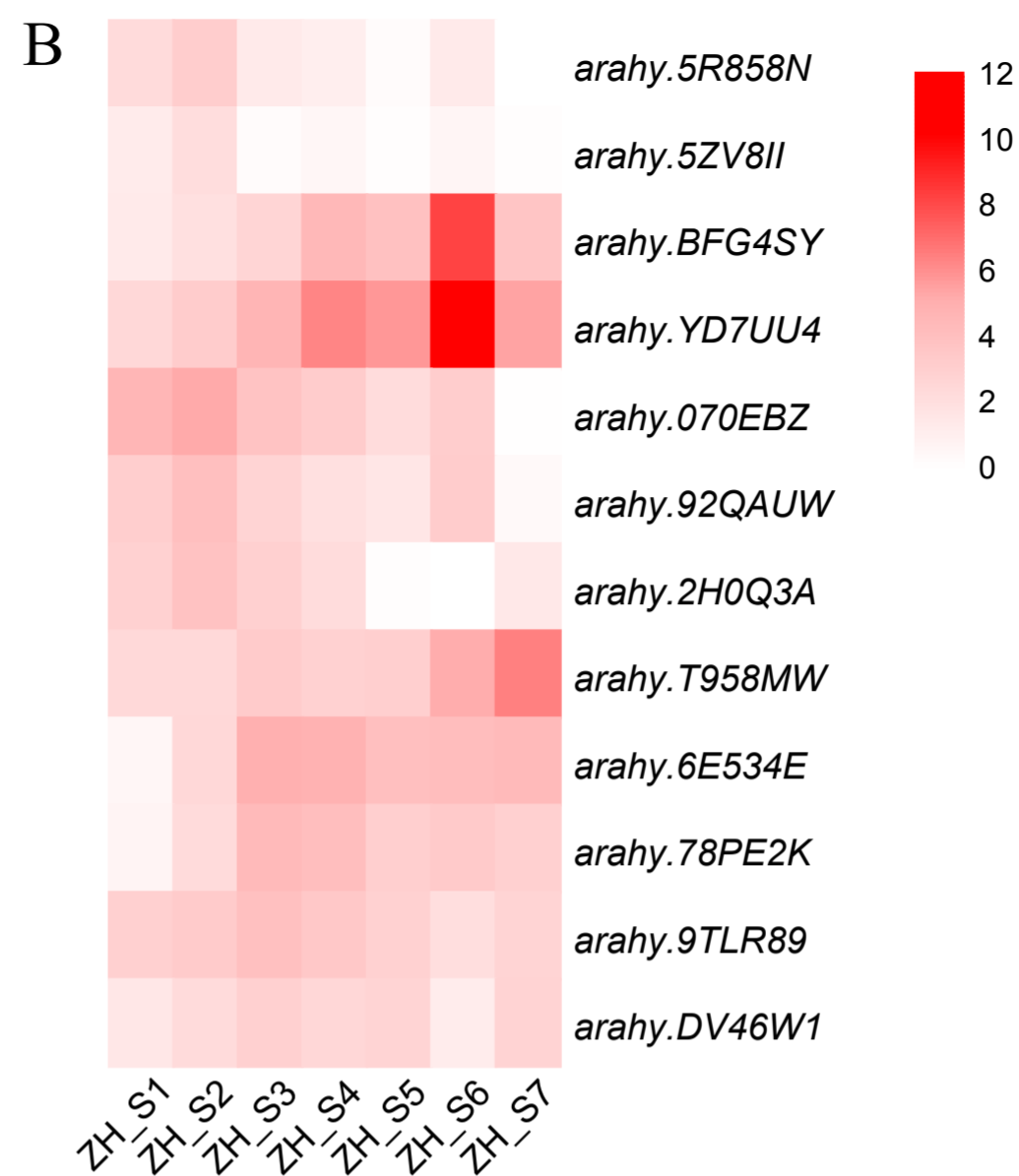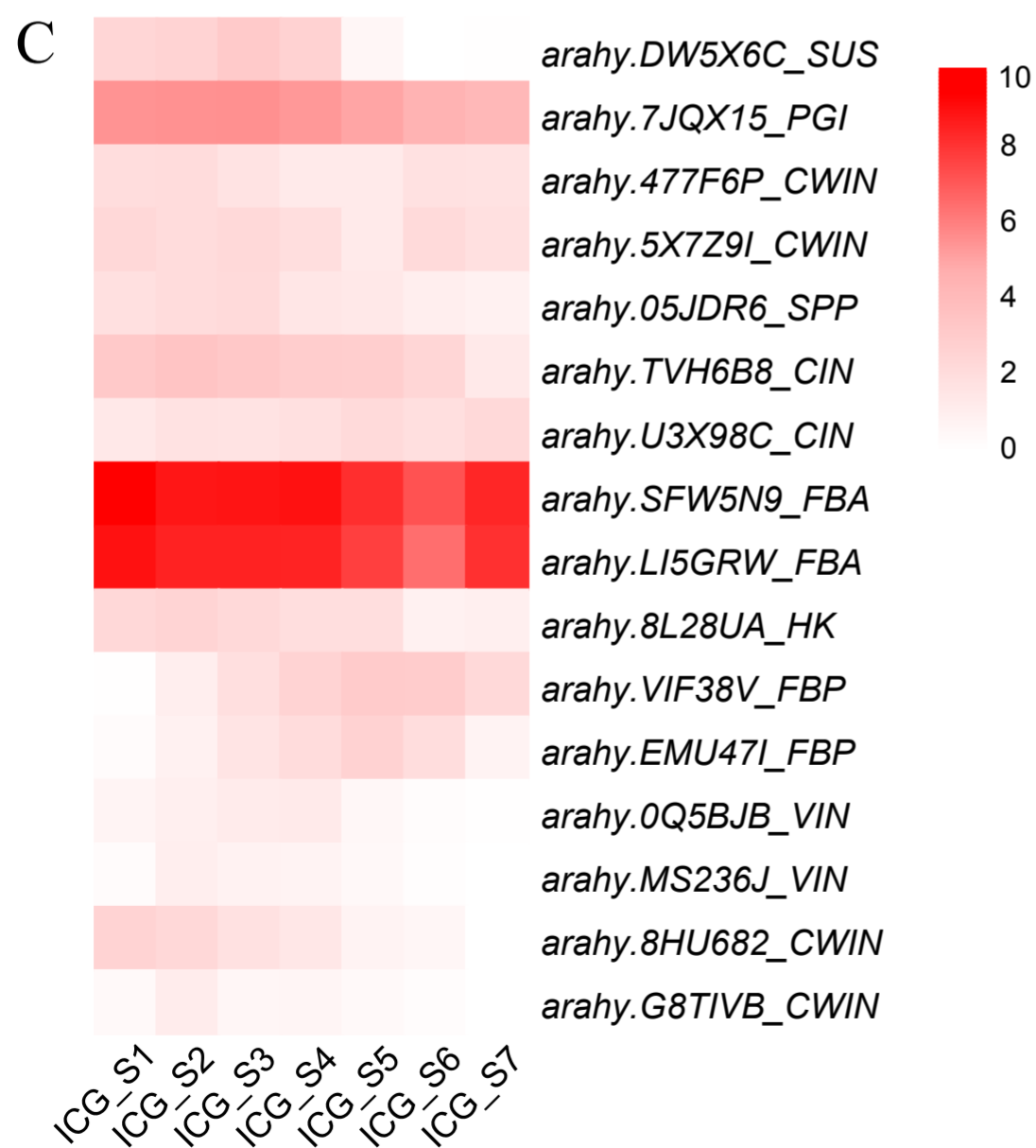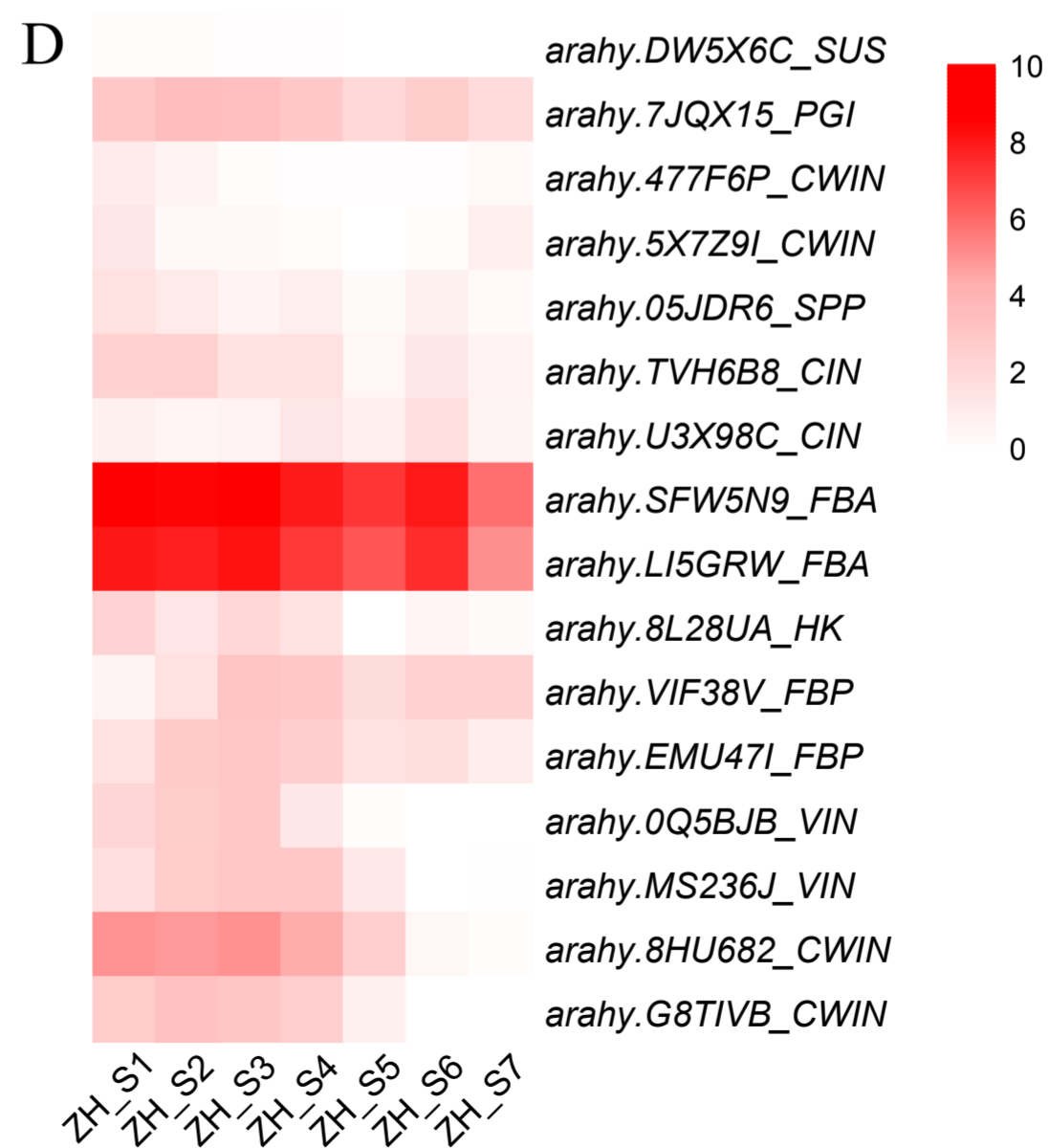

Supplement: Supplementary file 1 [file ijms-22-07266-s001.zip › ijms-1247925-supplementary/Supporting Information/Supplementary Figure S8.pdf]

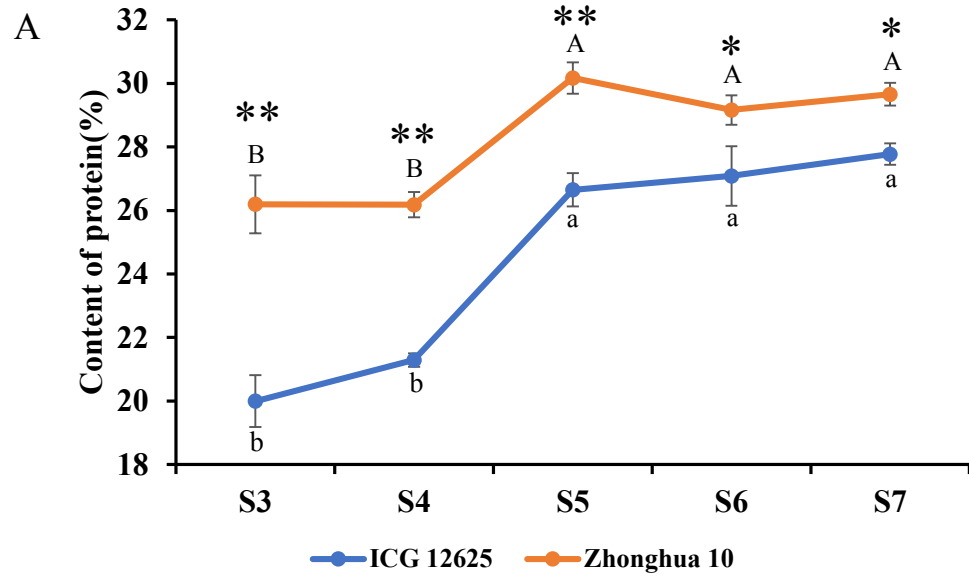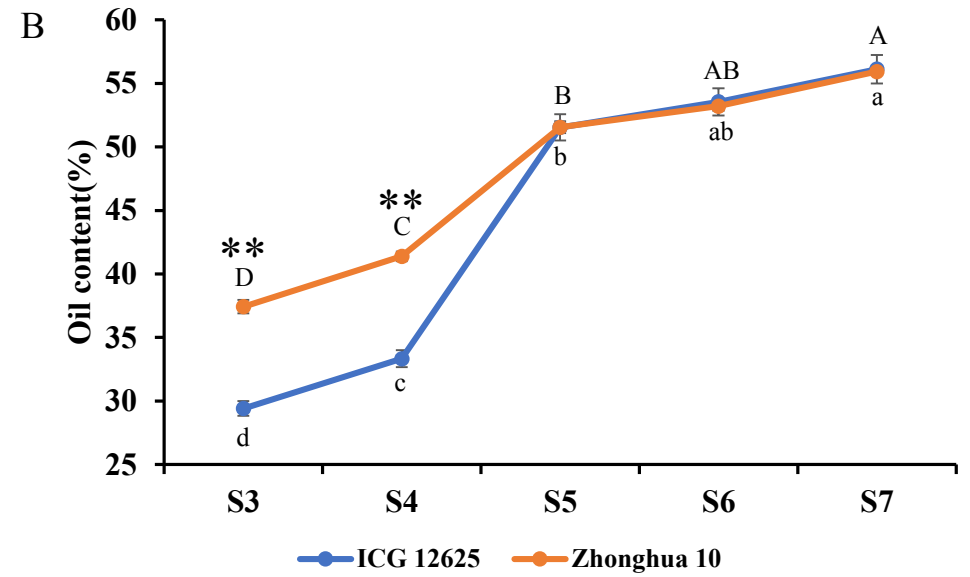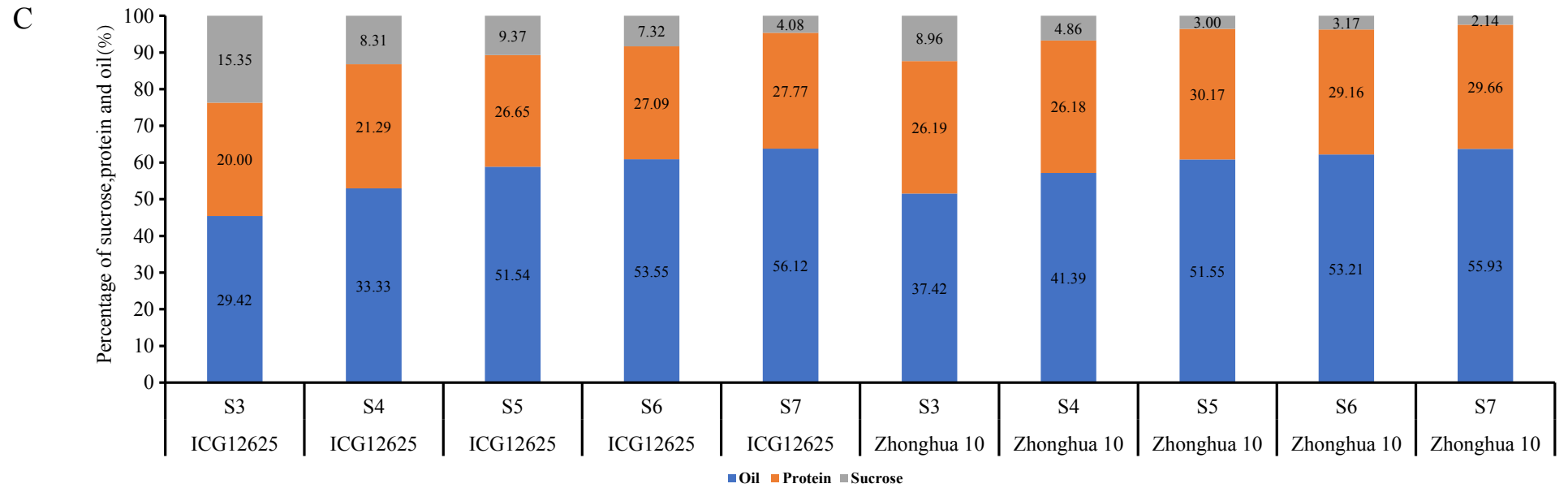

Supplement: Supplementary file 1 [file ijms-22-07266-s001.zip › ijms-1247925-supplementary/Supporting Information/Supplementary Figure S9.pdf]
